# Supplementary material for: Social care data and its fitness for integrated health and social care service governance: an exploratory qualitative analysis in the Dutch context
Source: BMJ Open. 2024 Apr 25;14(4):e078390. doi: 10.1136/bmjopen-2023-078390 (PMC11057269; doi:10.1136/bmjopen-2023-078390)
Supplement: Supplementary data [file bmjopen-2023-078390supp001.pdf]

Manuscript – Social care data and its fitness for integrated health and social care service governance: an exploratory qualitative analysis in the Dutch context

Véronique LLC Bos[1,2], Niek S Klazinga[1,2] and Dionne S Kringos[1,2]

[1] Department of Public and Occupational Health, Amsterdam UMC Location University of Amsterdam, Meibergdreef 9, Amsterdam, The Netherlands.

[2] Quality of Care, Amsterdam Public Health research institute, Amsterdam, The Netherlands.

Corresponding author

Véronique Bos

Department of Public and Occupational Health, Amsterdam UMC, University of Amsterdam

Van der Boechorststraat 7, 1081 BT Amsterdam, the Netherlands

Email: v.l.bos@amsterdamumc.nl

Supplemental Material 1 COREQ 32-item checklist

Domain 1: Research team and reflexivity

Personal Characteristics

Nr	Item	Guide question/description	Answer and where to find in manuscript
1	Interviewer/facilitator	Which author/s conducted the interview or focus group?	Interviews: VB<br><br>Reflection session: NK, DK and VB<br><br>Contributorship statement page 19
2	Credentials	What were the researcher's credentials? E.g. PhD, MD	VB: BSc, MA<br><br>NK: Full Professor, MD<br><br>DK: Associate Professor<br><br>Contributorship statement page 19
3	Occupation	What was their occupation at the time of the study?	VB: PhD candidate<br><br>NK: Professor of Social Medicine

			DK: Associate Professor and Principal Investigator & Educator<br><br>Contributorship statement page 19
4	Gender	Was the researcher male or female?	VB: female<br><br>NK: male<br><br>DK: female<br><br>Contributorship statement page 19
5	Experience and training	What experience or training did the researcher have?	Experienced in interviews, reflection sessions and qualitative research<br><br>Contributorship statement page 19

Relationship with participants

Nr	Item	Guide question/description	
6	Relationship established	Was a relationship established prior to study commencement?	Before the start of the study, the study and the research team were introduced to the interviewees and reflection session experts via email.<br><br>Research Methods pages 7-8
7	Participant knowledge of the interviewer	What did the participants know about the researcher? e.g. personal goals, reasons for doing the research	In the communication to participants the background of the interviewer was listed, as well as research goals.<br><br>Research Methods pages 7-8
8	Interviewer characteristics	What characteristics were reported about the interviewer/facilitator? e.g. Bias, assumptions, reasons and interests in the research topic	The credentials, occupation and department of the research team was communicated.<br><br>Research Methods pages 7-8

Domain 2: study design

Theoretical framework

Nr	Item	Guide question/description	
9	Methodological orientation and Theory	What methodological orientation was stated to underpin the study? e.g. grounded theory, discourse analysis, ethnography, phenomenology, content analysis	A constructivism explorative approach with inductive reasoning was used. Research Methods page 6

Participant selection

Nr	Item	Guide question/description	
10	Sampling	How were participants selected? e.g. purposive, convenience, consecutive, snowball	Purposive sampling based on the desktop research and participant referral Research Methods page 7
11	Method of approach	How were participants approached? e.g. face-to-face, telephone, mail, email	The participants were approached via email, phone and LinkedIn. Research Methods page 7
12	Sample size	How many participants were in the study?	18 participants in the interviews, and 10 participants in the reflection session. Results page 9
13	Non-participation	How many people refused to participate or dropped out? Reasons?	3 participants dropped out without stating a reason, 1 participant refused to participate due to prioritisation issues at that moment Research Methods page 7

Setting

Nr	Item	Guide question/description	
14	Setting of data collection	Where was the data collected? e.g. home, clinic, workplace	Most interviews were conducted digitally or in the work environment of the interviewee.

			The reflection session was held digitally.<br><br>Research Methods page 7
15	Presence of non-participants	Was anyone else present besides the participants and researchers?	No<br><br>Contributorship statement page 19
16	Description of sample	What are the important characteristics of the sample? e.g. demographic data, date	Participants from different levels of the health care system (micro, meso, macro) and different perspectives (clients, providers, data processors, policy advisors) were included in the sample.<br><br>Research Methods page 7

Data collection

Nr	Item	Guide question/description	
17	Interview guide	Were questions, prompts, guides provided by the authors? Was it pilot tested?	The interview guideline was grounded in the Health care performance intelligence pyramid and is provided in appendix 3. It was not pilot tested.<br><br>Research Methods page 7
18	Repeat interviews	Were repeat interviews carried out? If yes, how many?	No, however some questions that could not be answered during interviews were answered at a later moment in time or corrected in the transcript by the interviewee.<br><br>Research Methods page 8
19	Audio/visual recording	Did the research use audio or visual recording to collect the data?	Interviews and the reflection session were audio recorded<br><br>Research Methods page 8

20	Field notes	Were field notes made during and/or after the interview or focus group?	Notes were taken by two researchers (VB and DK) during the reflection session.<br><br>Contributor statement page 19
21	Duration	What was the duration of the interviews or focus group?	The duration of the interviews were between 33-60 minutes.<br><br>Research Methods page 7
22	Data saturation	Was data saturation discussed?	Participants could adjust content to their transcript without limitations and the reflection had the function to validate and if needed complement draft findings.<br><br>Research Methods pages 7-8
23	Transcripts returned	Were transcripts returned to participants for comment and/or correction?	Transcripts were emailed to the participants and they were given at least two weeks to review and adjust content to their transcript without limitations.<br><br>Results page 8

Domain 3: analysis and findings

Data analysis

Nr	Item	Guide question/description	
24	Number of data coders	How many data coders coded the data?	One researcher VB drafted the results and the reflection session was used to complement and validate principle findings.<br><br>Contributorship statement page 19
25	Description of the coding tree	Did authors provide a description of the coding tree?	No, a reflection session with experts was used to validate principle findings.<br><br>Research Methods page 8

26	Derivation of themes	Were themes identified in advance or derived from the data?	The transcript pieces were ordered by research question and themes were identified from these grouped transcript pieces.<br><br>Research Methods page 8-9
27	Software	What software, if applicable, was used to manage the data?	Excel was used to order and theme the transcript pieces.<br><br>Research Methods page 8
28	Participant checking	Did participants provide feedback on the findings?	Yes, the reflection session was used to complement and validate draft findings.<br><br>Research Methods page 8

Reporting

Nr	Item	Guide question/description	
29	Quotations presented	Were participant quotations presented to illustrate the themes / findings? Was each<br><br>quotation identified? e.g. participant number	One quote was used anonymously in the article and can be retraced by the researchers via the transcript identification number.<br><br>Results page 15
30	Data and findings consistent	Was there consistency between the data presented and the findings?	Yes, no contradicting statements were made in the interviews.<br><br>Results pages 9-16
31	Clarity of major themes	Were major themes clearly presented in the findings?	There was overall consensus in the reflection session on the final principal findings.<br><br>Results pages 9-16
32	Clarity of minor themes	Is there a description of diverse cases or discussion of minor themes?	In the reflection session examples were added to some statements to highlight nuances.<br><br>Results pages 9-16
