# Supplementary material for: Social care data and its fitness for integrated health and social care service governance: an exploratory qualitative analysis in the Dutch context
Source: BMJ Open. 2024 Apr 25;14(4):e078390. doi: 10.1136/bmjopen-2023-078390 (PMC11057269; doi:10.1136/bmjopen-2023-078390)
Supplement: Supplementary data [file bmjopen-2023-078390supp002.pdf]

Manuscript – Social care data and its fitness for integrated health and social care service governance: an exploratory qualitative analysis in the Dutch context

Véronique LLC Bos[1,2], Niek S Klazinga[1,2] and Dionne S Kringos[1,2]

[1] Department of Public and Occupational Health, Amsterdam UMC Location University of Amsterdam, Meibergdreef 9, Amsterdam, The Netherlands.

[2] Quality of Care, Amsterdam Public Health research institute, Amsterdam, The Netherlands.

Corresponding author

Véronique Bos

Department of Public and Occupational Health, Amsterdam UMC, University of Amsterdam

Van der Boechorststraat 7, 1081 BT Amsterdam, the Netherlands

Email: v.l.bos@amsterdamumc.nl

Supplemental Material 2: Dashboards desktop research & Data sources

Dashboards

Waar staat je gemeente (www.waarstaatjegemeente.nl)

Gemeentezorgspiegel (https://www.vektis.nl/gemeentezorgspiegel)

Regiobeeld (https://www.regiobeeld.nl/)

Labour Market Dashboards (https://www.werk.nl/arbeidsmarktinformatie/dashboards)

Early warning dashboard for possible debt (https://monitorvroegsignaleringshv.nl)

Data sources of included indicators

CBS - Arbeidsdeelname

CBS - Bevolkingsstatistiek

CBS - Bijstandsuitkeringenstatistiek

CBS - Enquête beroepsbevolking

CBS - Enquête sociale samenhang en Welzijn

CBS - Jeugdmonitor

CBS - Gezondheidsmonitor Volwassenen en Ouderen

CBS - Gemeentelijke Monitor Sociaal Domein

CBS - Monitor Abonnementstarief

CBS – Statistiek Re-integratie door Gemeenten

Divosa - Monitor Schuldhulpverlening

Divosa - Monitor Vroegsignalering Schulden

GGD-GHOR

RIVM

Vektis
