# Supplementary material for: Social care data and its fitness for integrated health and social care service governance: an exploratory qualitative analysis in the Dutch context
Source: BMJ Open. 2024 Apr 25;14(4):e078390. doi: 10.1136/bmjopen-2023-078390 (PMC11057269; doi:10.1136/bmjopen-2023-078390)
Supplement: Supplementary data [file bmjopen-2023-078390supp003.pdf]

Manuscript – Social care data and its fitness for integrated health and social care service governance: an exploratory qualitative analysis in the Dutch context

Véronique LLC Bos[1,2], Niek S Klazinga[1,2] and Dionne S Kringos[1,2]

[1] Department of Public and Occupational Health, Amsterdam UMC Location University of Amsterdam, Meibergdreef 9, Amsterdam, The Netherlands.

[2] Quality of Care, Amsterdam Public Health research institute, Amsterdam, The Netherlands.

Corresponding author

Véronique Bos

Department of Public and Occupational Health, Amsterdam UMC, University of Amsterdam

Van der Boechorststraat 7, 1081 BT Amsterdam, the Netherlands

Email: v.l.bos@amsterdamumc.nl

Supplemental Material 3 Translated interview guide

Thank you for your time

Going through informed consent form – when agreed, proceed

Affirm the scope of the research: Wet Maatschappelijke Ondersteuning, Participatiewet, Wet Gemeentelijke Schuldhulpverlening

- What data do you collect?
  - What is the purpose of your data collection?
  - Is there underlying legislation, standardization or other agreements or confinements as to how to manage the data?
- Data input points (potential legislation/standardization at entry point)
  - Who does the entry of the data?
  - With what purpose is the data entered in the system?
  - Is there underlying legislation, standardization or other agreements or confinements as to how to enter the data?
- Data sources and custodians
  - What database is the data collected in and who is owner of the database?
- Interoperability of sources
  - Does the database interact with other databases? If yes, with which other databases?

- Data management responsibilities, stakeholder inclusion, and regulators
    - How is data quality managed?
    - Are stakeholders included in quality management, transferring data to performance indicators, or other ways of data management?
    - What internal/external controls are executed for the database?
- Data outputs (information) and its users
    - What indicators are presented from the database?
    - Who uses the indicators to inform their decision making?
- In your perspective how can we make good use of social care data within the three laws mentioned for the benefit of integrated care? And what are potential hurdles to make use of this data?
