# Supplementary material for: Social care data and its fitness for integrated health and social care service governance: an exploratory qualitative analysis in the Dutch context
Source: BMJ Open. 2024 Apr 25;14(4):e078390. doi: 10.1136/bmjopen-2023-078390 (PMC11057269; doi:10.1136/bmjopen-2023-078390)
Supplement: Supplementary data [file bmjopen-2023-078390supp004.pdf]

Manuscript – Social care data and its fitness for integrated health and social care service governance: an exploratory qualitative analysis in the Dutch context

Véronique LLC Bos[1,2], Niek S Klazinga[1,2] and Dionne S Kringos[1,2]

[1] Department of Public and Occupational Health, Amsterdam UMC Location University of Amsterdam, Meibergdreef 9, Amsterdam, The Netherlands.

[2] Quality of Care, Amsterdam Public Health research institute, Amsterdam, The Netherlands.

Corresponding author

Véronique Bos

Department of Public and Occupational Health, Amsterdam UMC, University of Amsterdam

Van der Boechorststraat 7, 1081 BT Amsterdam, the Netherlands

Email: v.l.bos@amsterdamumc.nl

Supplemental Material 4 Interviewee list with background/expertise

organization	WHY?
VNG GMSD	Custodian of the Municipal Monitor of the Social Domain Dashboard and data.
VNG voorspelmodel	Custodian of the Social Care Act (WMO) prediction model
Divosa	Knowledge organisation supporting municipalities
Municipality of Amsterdam	Data team of the municipality
Stichting Inlichtingenbureau	Data processing service organisation established by the Ministry of Social Affairs and Employment (SZW) processing data about citizens who use social care schemes and facilities.
Vektis	Custodian of the Gemeentezorgspiegel (municipal care dashboard)
De Friesland	Health care insurer in the Friesland region working on integrated care projects using data.
RIVM (Regiobeeld)	Custodian of Regiobeeld

Sociaal Werk Versterkt	Representative of social workers
De Sociale Maatschap	Social care provider in de Amsterdam Noord region
Ketenbureau i-sociaal Domein	Data processing service organisation which facilitates and supports municipalities and healthcare providers in, among other things, simplifying the process from purchasing and tendering to accountability and reducing administrative burdens.
ZorgInstituut	Custodians of the i-standards (e.g. i_WMO standard)
BKR	Custodian credit registry (BKR)
LCR	National representatives for clients in social care
