# Supplementary material for: Social care data and its fitness for integrated health and social care service governance: an exploratory qualitative analysis in the Dutch context
Source: BMJ Open. 2024 Apr 25;14(4):e078390. doi: 10.1136/bmjopen-2023-078390 (PMC11057269; doi:10.1136/bmjopen-2023-078390)
Supplement: Author's manuscript [file bmjopen-2023-078390.draft_revisions.pdf]

BMJ Open is committed to open peer review. As part of this commitment we make the peer review history of every article we publish publicly available.

When an article is published we post the peer reviewers' comments and the authors' responses online. We also post the versions of the paper that were used during peer review. These are the versions that the peer review comments apply to.

The versions of the paper that follow are the versions that were submitted during the peer review process. They are not the versions of record or the final published versions. They should not be cited or distributed as the published version of this manuscript.

BMJ Open is an open access journal and the full, final, typeset and author-corrected version of record of the manuscript is available on our site with no access controls, subscription charges or pay-per-view fees (http://bmjopen.bmj.com).

If you have any questions on BMJ Open's open peer review process please email info.bmjopen@bmj.com

Social care data and its fitness for integrated health and social care service governance: an exploratory qualitative analysis in the Dutch context

Journal:	*BMJ Open*
Manuscript ID	bmjopen-2023-078390
Article Type:	Original research
Date Submitted by the Author:	31-Jul-2023
Complete List of Authors:	Bos, Véronique; Amsterdam UMC Location AMC, Department of Public and Occupational Health; Amsterdam Public Health Research Institute, Quality of Care<br>Klazinga, Niek; Amsterdam UMC Locatie AMC, Department of Public and Occupational Health; Amsterdam Public Health Research Institute, Quality of Care<br>Kringos, D; Amsterdam UMC Location AMC, Department of Public and Occupational Health; Amsterdam Public Health Research Institute, Quality of Care
Keywords:	Information management < BIOTECHNOLOGY & BIOINFORMATICS, Health policy < HEALTH SERVICES ADMINISTRATION & MANAGEMENT, Organisation of health services < HEALTH SERVICES ADMINISTRATION & MANAGEMENT, Information Extraction < Information Storage and Retrieval, QUALITATIVE RESEARCH, SOCIAL MEDICINE

SCHOLARONE™
Manuscripts

I, the Submitting Author has the right to grant and does grant on behalf of all authors of the Work (as defined in the below author licence), an exclusive licence and/or a non-exclusive licence for contributions from authors who are: i) UK Crown employees; ii) where BMJ has agreed a CC-BY licence shall apply, and/or iii) in accordance with the terms applicable for US Federal Government officers or employees acting as part of their official duties; on a worldwide, perpetual, irrevocable, royalty-free basis to BMJ Publishing Group Ltd ("BMJ") its licensees and where the relevant Journal is co-owned by BMJ to the co-owners of the Journal, to publish the Work in this journal and any other BMJ products and to exploit all rights, as set out in our licence.

The Submitting Author accepts and understands that any supply made under these terms is made by BMJ to the Submitting Author unless you are acting as an employee on behalf of your employer or a postgraduate student of an affiliated institution which is paying any applicable article publishing charge ("APC") for Open Access articles. Where the Submitting Author wishes to make the Work available on an Open Access basis (and intends to pay the relevant APC), the terms of reuse of such Open Access shall be governed by a Creative Commons licence – details of these licences and which Creative Commons licence will apply to this Work are set out in our licence referred to above.

Other than as permitted in any relevant BMJ Author's Self Archiving Policies, I confirm this Work has not been accepted for publication elsewhere, is not being considered for publication elsewhere and does not duplicate material already published. I confirm all authors consent to publication of this Work and authorise the granting of this licence.

For peer review only

Social care data and its fitness for integrated health and social care service governance: an exploratory qualitative analysis in the Dutch context

Véronique LLC Bos[1,2], Niek S Klazinga[1,2] and Dionne S Kringos[1,2]

[1] Department of Public and Occupational Health, Amsterdam UMC Location University of Amsterdam, Meibergdreef 9, Amsterdam, The Netherlands.

[2] Quality of Care, Amsterdam Public Health research institute, Amsterdam, The Netherlands.

Corresponding author

Véronique Bos

Department of Public and Occupational Health, Amsterdam UMC, University of Amsterdam

Van der Boechorststraat 7, 1081 BT Amsterdam, the Netherlands

Email: v.l.bos@amsterdamumc.nl

Supplementary material

Supplemental Material 1: COREQ 32-item checklist for this study

Supplemental Material 2: Sources desk top research

Supplemental Material 3: Translated interview guide

For peer review only

Abstract (293 words)

INTRODUCTION: To date, little is known on how social care data could be used to inform performance-based governance to accelerate progress towards integrated care.

OBJECTIVES AND DESIGN: This study aims to perform a descriptive evaluation of available social care data in the Netherlands and its fitness for integrated health and social care service governance. An exploratory mixed method qualitative study was undertaken based on desktop research (41 included indicators), semi-structured expert interviews (thirteen interviews including eighteen experts) and a reflection session (ten experts).

SETTING: The Netherlands; social care is defined as care provided in accordance with the Social Support Act, the Participation Law, and the Law for Municipal debt-counselling.

RESULTS: This study found six current uses for social care data: 1) communication and accountability, 2) monitoring social care policy, 3) early warning systems, 4) controls and fraud detection, 5) outreaching efforts, and 6) prioritisation. Further optimization should be sought through 4 directions: standardization, management of data exchange across domains, awareness of the link between registration and financing, strengthening the overall trust in data sharing. The study found five potential uses for social care data to improve the governance in integrated care: 1) cross-domain learning and cooperation (e.g. through benchmarks), 2) preventative measures and early warning systems, 3) give insight regarding the quality and effectivity of social care in a broader perspective, 4) clearer accountability of social care towards contracting parties and policy, 5) enable cross-sector data-driven governance model.

CONCLUSION: Although there are several innovative initiatives for the optimization of the use of social care data in the Netherlands, the current social care data landscape and management is not yet fit to support the new policy initiatives to strengthen integrated health and social care service governance. Four directions for addressing the shortcomings are provided.

For peer review only

Strengths and limitations

- In our desk-top research we used an explorative approach and publically available data sources. This can have biased our findings towards finding more data sources aligned with government roles.
- We limited our scope to data sources with a national representation which can have biased results towards finding more aggregate data.
- We minimized these biases by including experts from different levels (micro, meso and macro) and experts from a diverse social care data processing and use background in the interviews and reflection session to enrich the desktop study and validate results.

- Public sources in social care data like the dashboards and monitors used in this study are constantly developing, thus the results of the study are subject to change over time.

Keywords (3-6)

social care data, performance information, Integrated health services, the Netherlands, informed decision making, health care governance

For peer review only

Introduction (background & problem statement)

Sound and reliable health and social care information at the right place and at the right time is essential for effective decision making in health systems (Colombo, Oderkirk, & Slawomirski, 2020; Kringos et al., 2020; World Health Organization, 2010). Integrated health services, described by the WHO as "health services that are managed and delivered in a way that ensures people receive a continuum of health promotion, disease prevention, diagnosis, treatment, disease management, rehabilitation and palliative care services, at the different levels and sites of care within the health system, and according to their needs throughout their life course"(WHO, 2015), has become an important focus of many health system reforms. As a consequence, performance information presented in the traditional siloes of public health data, health care data and social care data form a hindrance for effective governance across the domains of health promotion, disease prevention, diagnosis, treatment, disease management, rehabilitation and palliative care services (OECD, 2020a, 2020b).

The COVID-19 pandemic has highlighted that in order to effectively monitor and govern our health systems, data from a broad range of sources using demographics, social care data, health care data, and public health data needs to be systematically integrated. This is demonstrated by the need for a dual-track health system management (monitoring specific and generic information in coherence) and including social, behavioural and economic perspectives next to epidemiologic considerations (World Health Organization, 2020). Countries worldwide are struggling with integrating these diverse types of data to establish comprehensive performance intelligence for decision making. Despite data rich health systems, these systems are information poor, indicating an unfulfilled potential of data and digital technologies (OECD, 2019). Additionally, the COVID-19 pandemic has increased the pressure on the often already overstretched health care services and national budgets globally (OECD, 2021). In order to reduce this pressure policy makers are shifting their attention to health promotion and disease prevention. Strengthening social care is part of this agenda. Investments in social care (e.g. housing, income support, nutrition support, care coordination, and community outreach) can have a positive impact on the health of citizens and health care service needs and use (Taylor et al., 2016).

Countries and regions worldwide are investing in an integrated health and social care data infrastructure. The main purposes of the data infrastructures differ from primary uses of data (e.g. supporting clinical practice) to facilitate a vast range of secondary data uses (e.g. research, policy, governance) on regional or national levels. For instance, Finland's Sotkanet, a national health and social care data infrastructure, has more comprehensive data available to inform their decision making from

For peer review only

a broader welfare perspective (Kärki & Ailio, 2014). The affiliated Oulu Self Care Service shows that an integrated data system can also function as an enabler and improve chronic care health outcomes and efficiency through shared use of health and social care data among care providers (Centre et al., 2015). New Zealand's integrated health infrastructure (IDI) illustrates that cross-cutting data can give insights needed to govern effectively throughout the health and welfare system (Milne et al., 2019; Zhao, Gibb, Jackson, Mehta, & Exeter, 2018). This national integrated health infrastructure was for example used for descriptive research to characterize adolescents who experience poor health outcomes (McLeod, 2015). It was also used to define cohorts in the existing population; one example was a cohort of people with chronic diseases to define effects on work and income (Dixon, 2015). Thus, linking social care data to health care data shows potential for integrated governance, but there are also known pitfalls. A Canadian study linking administrative social assistance data to health care data warns for potential biases due to linkage errors (de Oliveira et al., 2022). A study in Dundee, Scotland found that linkage between health and social care data faced challenges around data linkage (e.g. use of a shared identifier across organisations), data analysis (e.g. understanding missing data), and the need for tacit knowledge (e.g. people understanding the data, it's limitations and what it represents) limiting the full exploitation (Witham, Frost, McMurdo, Donnan, & McGilchrist, 2015). The Integrated Children's System in the UK initially followed a top-down managerial approach emphasizing on accountability of institutional risk instead of user-centeredness which had a detrimental effect on professional autonomy (Wastell & White, 2014). These examples show that a thorough understanding of social care data, its characteristics and use are needed to exploit its potential to gain actionable insights.

In the Netherlands multi-stakeholder policy agreements on strategic directions are considered a classical tool for governing the health- and welfare sectors. In 2022 Dutch stakeholders in the health and welfare sector sectors launched multiple integrated policy agreements and programmes to facilitate a more integrate delivery of health and social care services: the Integrated Care Agreement (IZA), the Healthy and Active living Agreement (GALA), the Housing, Support and Care for the Elderly Programme (WOZO) and the Future-proof Care and Welfare Labour Market Programme (Rijksoverheid, 2022a, 2022b, 2022c, 2022d). In these initiatives, cooperation and integration of health and social care services and prevention efforts are highlighted as important ways forward to subdue the pressure on the healthcare system and its staff. The Netherlands has many (N: > 450) care partnerships (Partners, 2022). These are often regional initiatives and alliances that are strengthening their provision of integrated health and care services for better outcomes (Bos, Klazinga, & Kringos, 2021). The bandwidth of data-driven action in these collaborations is broad. Some collaborations see it as a need to have, others as a nice to have (Partners, 2022). With a high political and regional interest

For peer review only

in integrating health and social services, the Netherlands is a relevant country for an explorative study on social care data and its fitness for integrated health and social care service governance.

General aims and objectives

This study aims to perform a descriptive evaluation of the landscape of social care data in the Netherlands, its current use and how it can be improved. It attempts to explore the fitness for purpose and use of social care data to contribute to integrated health and social care service governance.

Research questions

1. What social care data is available in the Netherlands? What is its current use and how can the social care data be improved to serve integrated health and social care service governance?
2. How can social care data potentially be used to improve integrated health and social care service governance?

Research Methods

Research design and scope

An exploratory qualitative study design (Rendle, Abramson, Garrett, Halley, & Dohan, 2019) was employed using: 1) desktop research, 2) semi-structured expert interviews and 3) a reflection session. The study conforms to the Consolidated Criteria for Reporting Qualitative Studies, the 32-item checklist for this study can be found in Supplemental Material 1. Our research design was grounded in the Health care performance intelligence pyramid (Kringos et al., 2020). The hierarchical pyramid defines how measurement through data collection and its translation to indicators is at the base of governance and management through information and knowledge. Action and utilisation is the ultimate end goal and needs its own translation from information and knowledge to practice. Each of these steps needs to consider the regulatory, organisational, political and cultural context.

The scope of this research is social care as provided within the municipal responsibilities in the Netherlands through: 1) the Social Support Act (Wet Maatschappelijke Ondersteuning, WMO); regulating municipal responsibility for supplementing citizens in their care need, 2) the Participation Law (Participatiewet); regulating municipal responsibility for supporting citizens in participating in the society, and 3) the Law for Municipal debt-counselling (Wet Gemeentelijke Schuldhulpverlening); regulating municipal responsibility for supporting citizens in financial need. As the focus of this study is on the adult population we have excluded social care as provided by the Youth Act (Jeugdwet);

For peer review only

regulating the preventative and mental health care for children. As social care was decentralised to municipal level in 2015 in the Netherlands, we have only included documents from beyond this time point in our desktop research.

Data collection and coding

Our data collection took place from January 2022 to October 2022. The desktop research aimed to get an overview of publicly available social care indicators. We used two starting points: 1) the website vzinfo.nl of the National Institute for Public Health and the Environment commissioned by the Ministry of Health, Welfare and Sport, which provides an overview and metadata of available public health and care information and 2) a social care stakeholder analysis done by Driesten and Blink in 2021 (Van Driesten, 2021). Data included in this study was either collected on a national level, or purposefully sampled to represent the national population. Data(sets) that were collected only for a single organisation/municipality or region were excluded from this study. We collected all described indicators from dashboards and reports found via these two starting points that met the scope of this research. An overview of all the collected dashboards and reports included in the desktop research and their references can be found in Supplemental Material 2.

The expert interviews aimed to complement the desktop research and highlight current uses of social care data and indicators within the social care domain, to describe ways to optimize social care data (infrastructure), and to highlight potential uses for integrated health and social care service governance. We purposively sampled experts based on the sources found in the desktop research, and gave them the opportunity to refer to other experts in the field of social care data in the Netherlands. We aimed to have at least two representatives from each level of the health care system: micro (social care clients and care professionals), meso (data custodians and data processing organizations), and macro (policy and legal experts) levels. The interview guideline (Supplemental Material 3) was grounded in the Health care performance intelligence pyramid. With the informed consent of participants, all interviews were audio-recorded and transcribed. Transcripts were emailed to the participants and they were given at least two weeks to review and adjust the content of their transcript without limitations. The validated transcripts were used for the thematic analysis.

The reflection session was organized on 12 October 2022 with the following aims: 1) to validate the draft results from the desktop research and semi-structured interviews and 2) to consolidate key findings. It was organized digitally in the aftermath of the COVID-19 pandemic. First, the study team presented the draft results. Second, participants shared their general reflections on the draft results. Finally, the following questions were discussed in-depth: 1) Is the outlined landscape of available social

For peer review only

care data and indicators, its management and its use recognisable?, 2) How can we optimize the use of available social care data within the social care domain?, 3) How can social care data contribute to public health & health care? And vice versa?, and 4) How can social care data be used in governing towards regional integrated care provision? The reflection session was audio recorded with the informed consent of all participants. The draft results from the desktop study and expert interviews were shared with the participants in advance.

Data analysis

To answer research question one, we used information retrieved from desktop research and interviews. First we constructed an overview of all dashboards and documents collected with their data custodian and a qualitative description of their content (Supplemental Material 2). Then we abstracted all indicators within the scope of this research and grouped them in their legal silos. Finally we categorized them into the following subgenres: input, process and output, outcome, and impact and allocated source types (administrative, medical record, registry, survey, direct observation) to each indicator. Transcript pieces that described available social care data in the Netherlands were used to enrich the desktop research by adding additional data sources. We selected and then grouped all pieces of the transcripts from the interviews that described social care data in the Netherlands, its current use and how it can be improved. Then we analysed the transcript pieces using an analytic induction process using key words and statements to define how social care data is currently used and can be improved.

To answer research question two, pieces of the interview transcripts were grouped that relate to the current and potential use of social care data to improve integrated health and social care service governance. Then an analytic induction process using key words and statements was used to define how social care data was used for integrated health and service governance. The draft results of research question one and two were presented, enriched, clarified and verified in the reflection session.

Results

Characteristics of the data sources and informants

The sources included in the desktop research can be found in Supplemental Material 2. Thirteen interviews were conducted with a total of eighteen experts including representatives for social care clients (n=1) and providers (n=2), representatives from the municipalities (n=2), social care administrative data processors (n=5, from 4 different organizations and working with different social

For peer review only

care data), owners of social care dashboards and indicator reports (n=6, from 4 different organizations) and experts from the health care insurance data field with experience in integrated care projects (n=2). The reflection session included ten experts on social care data, including representatives for social care clients (n=1) and providers (n=2), representatives from the municipalities (n=2), social care administrative data processors (n=4, from 3 different organizations and working with different social care data), and an expert from the health care insurance data field with experience in integrated care projects (n=1).

What social care data is available in the Netherlands and how is it used?

Measuring social care data: data collection and Indicators

The most common data source types used to populate the included social care indicators were administrative data (including the needs assessment done for administrative purposes) and surveys (often samples representative to national population). Indicators on use and costs of services were most common and most often reported on municipal or neighbourhood level. These indicators were often updated once or twice a year. There is a diversity in measurement instruments to measure an individual's needs for social care. However there is a national guideline for a needs assessment obligatory in order to receive the social care provided. Prediction models (e.g. the Social Support Act prediction model) are available, but in the interview with an expert on the prediction models, it was mentioned that policymakers are still hesitant to use prediction modelling to inform their policy actions.

The use of client reported data for decision making is very limited in the social care domain. There is a legal obligation in the Social Support Act for municipalities to carry out a national client experiences measure. Until 2021 this measure consisted of 10 nationally defined questions divided in three themes: 1) access to services, 2) quality of the services provided, and 3) the effect on the independence for the client and his/her participation in society. However since 2021, due to suboptimal use of the measure, municipalities are free to adapt the client experience measure to their needs, either using the 10 defined questions, adding additional questions or using their own survey format. This tailoring of the survey by municipalities might be detrimental to cross-municipality comparisons. Besides the national client experience measure, client stories are occasionally used to represent clients' voices to complement the data provided, this is relatively customary in the municipal reporting on social care.

The tables below give an overview of all included indicators and their defined indicator and data source type per legal silo.

For peer review only

Table 1 Social care indicators included in the desktop study within scope of the Social Support Act

**Indicator**	**Type of indicator**	**Type of source**
Trend in the number of potential Informal carers	(potential) Input	survey data
% Vulnerable population	(potential) Input	administrative / survey / medical record data
% of people with one or more limitation in daily functioning	(potential) Input	survey data
% of people with one or more restrictions in their mobility	(potential) Input	survey data
% of people with restrictions in their eye sight	(potential) Input	survey data
% of people with hearing disabilities	(potential) Input	survey data
Budgeted and actual costs for Social Support Act	Input	administrative
Municipal policy (only customized provisions, mainly customized provisions, as many general as customized provisions, mainly general provisions, only general provisions) of Social Support Act care provision	Input	survey data
Ratio between households with and without care provision	Process & Output	administrative
Relative number of households with provisions of care and support by number of provisions	Process & Output	administrative
Relative number of households with provisions of care and support by number of provisions per domain	Process & Output	administrative
Trend number of inhabitants with Social Support Act care provision	Process & Output	administrative
Relative number of inhabitants with Social Support Act care provision by category	Process & Output	administrative
Number of inhabitants with Social Support Act care provision	Process & Output	administrative
Relative number of inhabitants in sheltered housing by region	Process & Output	survey data / administrative
Number of terminated Social Support Act provisions by reason (total, deceased, planned/anticipated termination, move to other municipality, other/unknown)	Process & Output	administrative
Qualitative reporting of the WMO Client experience outcomes	Outcome	survey data

Table 2 Social care indicators included in the desktop study within scope of the Participation law

**Indicator**	**Type of indicator**	**Type of source**
Trend in pension age	Input	survey data
Ratio between households with and without care provision	Process & Output	administrative
Relative number of households with provisions of care and support by number of provisions	Process & Output	administrative
Relative number of households with provisions of care and support by number of provisions per domain	Process & Output	administrative
Number of inhabitants with a welfare benefit	Process & Output	administrative
Number of inhabitants with a welfare benefit by municipality and reference group	Process & Output	administrative
Number of inhabitants with a reintegration facility	Process & Output	administrative

For peer review only

Relative number of inhabitants with a reintegration facility by municipality and reference group	Process & Output	administrative
Number of unemployed inhabitants in working age	Outcome	survey data
% inhabitants participating in formal work, volunteer work and informal care by sex	Outcome	survey data
% of persons with/without health problems with formal work by age categories	Outcome	survey data
% of the Formal work population with/without health problems by age categories	Outcome	survey data
% of net formal work participation by age category and sex	Outcome	survey data
Number of inhabitants of the formal workers working part-time by sex	Outcome	survey data
% Unemployment by age category and sex	Outcome	survey data
Trend % net formal work participation by two age categories and sex	Outcome	survey data
Trend % unemployment by age category and sex	Outcome	survey data
% Unemployed young professionals	Outcome	survey data
SES-WOA score (financial prosperity, level of education and recent employment history of private households)	Impact	administrative

Table 3 Social care indicators included in the desktop study within scope of the Law for Municipal debt-counselling

**Indicator**	**Type of indicator**	**Type of source**
Municipal capacity to provide debt counselling services (enough capacity for demand, enough capacity for demand, but sustainability for the future is under pressure, not enough capacity for demand)	Input	survey data
Factors that determine the manner in which early warning signals for over indebtedness are followed up (type of notification, debt size, type of non-payment, age, composition of the household, time passed since notification, postal code)	Input	administrative / survey data
Number of applications for municipal debt counselling services	Process & Output	survey data
Qualitative reporting by cooperating municipalities on the (change in) target group for municipal debt counselling services	Process & Output	survey data
Number of early warning signals (one non-payment from one specific provider on one home address) per 1000 inhabitants per municipality	Process & Output	administrative
Number of early warning notifications (multiple signals at one address) per municipality	Process & Output	administrative
Number of contacts (defined as contact with a reaction, e.g. reply to email, opening the door, answering the phone call) resulting from early warning signals	Process & Output	administrative
% of inhabitants accepting the offer for debt counselling services	Process & Output	Administrative

For peer review only

SES-WOA score (financial prosperity, level of education and recent employment history of private households)	Impact	administrative

Governance and management in social care data: rules for translation to information and knowledge

Municipalities have the legal mandate for the social care provision. This mandate also includes how to structure the (administrative) social care data. This makes national level benchmarking between municipalities difficult, due to a high variability in interpretation and use of the available national product codes. National administrative registration standards are in place, for example the i-WMO standard, however diversity of payment models within and across municipalities make this data only interpretable: 1) within a known context (for example on municipal level) or 2) on a national level when product groups are aggregated to general terms. Our sources also showed a big variety between municipalities in data processing power, ranging from own data processing departments to the minimal data processing obligatory by law and for accountability purposes. This variety also exists between social care provider organizations.

Legislation on social care data use mainly covers accountability for the use of social provisions (fraud and supervision), use of the citizen service number for identification, and permission required for use of personal data (privacy). Multiple public and private parties can fulfil the role of data processor in social care in the Netherlands, often under municipal authority. But the use of social care data for quality improvement of care provision by integrated provider networks or policy action is not defined in the legislation. Thus a weighing, between respecting privacy of personal data on the one hand and having data available to deliver adequate social care services or take timely policy action on the other hand, cannot be made.

Utilisation of social care data: what is done with the acquired knowledge through data

Social care data is mostly used in the *communication and accountability* between care providers and financers (e.g. official needs assessments or registration of start and end a care trajectory), and in *monitoring social care policy* on municipal and national levels (e.g. Monitor on adult health and health of the elderly). There is an *early warning system* on over-indebtedness using non-payments of housing, utilities and health care insurance to alert municipalities to a potential demand in social care services to prevent over-indebtedness, according to one of the care organisations interviewed this information exchange has resulted in reduced forced out-of-house placements in the served district. However, other experts interviewed did highlight that in many regions the step between having data available and to use the data to identify and undertake the required actions by municipalities and

For peer review only

care providers to have an impact still leaves room for improvement. Data alone does not lead to action.

Traditionally, data in the social care domain has been used for *controls and fraud detection*, which has led to a distrust in data exchange in the social care domain from the care provider and client perspective. Examples of information used for controls and fraud detection mentioned by interviewees were: 1) financial information on care providers to guide the municipal purchasing risk management strategy and 2) information on which clients have access to Long Term Care Act resources to check whether the Social Support Act resources given are still relevant. Some recent examples rethink this approach, like the *outreaching efforts* of the Social Insurance Bank (SVB) to monitor and find citizens with the rights to receive a pension, who have for an unknown reason not (yet) requested it. We have also found a *prioritisation* example of a social work organization using national demographic data compared with their client base to assess if they are targeting the neighbourhood with the most (predicted) need for debt counselling.

Improving the social care data use

Data: Standardization of registration

A high variation between municipalities in social care data, data infrastructure and processing is the result of policy decentralization efforts aiming to provide care 'closer to home'. Efforts to standardize product codes have already yielded results (from 100.000 product codes in 2015 to about 3.000-4.000 product codes per law during the time of this research). However, some experts interviewed believe that more standardization in social care data, it's infrastructure and its processing can increase comparability and decrease administrative burden on the long run for care provider organizations and municipalities alike. We found heterogeneous roles and responsibilities in the coordination and alignment of data processing in social care. It has become difficult to compare even similar indicators due to source and processing variability. A clear governance structure in social care data, its infrastructure and processing could improve the consistency in translating the available data to its use. Standardization efforts can give a clear framework how to register data and reduce interpretation differences of data registration. It should be taken into account that standardizing data registration does not mean that regional variability in care delivery has to be reduced when different regions have different care needs.

Governance and management of social care data across domains and within clear roles for a societal purpose

Data processing is often operationalized per legal and financial silo. Experts state that in order to have a clear picture of the societal needs and impact of social care, insight in performance

information across legal and financial silos is needed (e.g. the association between over over-indebtedness and health care use). However, data use across domains is legally and politically restricted in the Netherlands. Additionally, cross-sector data sharing, including across private and public interests, may have more potential for *preventative measures*, but is politically sensitive. For example in debt counselling efforts (nationally organized) study loans consist of a higher average credit per person than consumer credits, however study loans, in comparison to consumer credits are not as transparently monitored for credit providers due to political sensitivity. These additional options for prevention could have effects if used with the necessary boundaries of an adequate ethical and legal framework to avoid discrepancies between data use and its societal purpose.

Legislation on data exchange and processing should emphasise the explicit use and purpose instead of limiting (and at the same time facilitating abundant) data processing and integration within the financial or legal silo. Measuring effectiveness and affordability of services needs emphasizing If the purpose is to help citizens in a different way, measurements need aligning to this new purpose. Data on the healthy lives of citizens, and how to maintain these lives, or how to signal when healthy lives are at risk, is, for example, currently limited.

Awareness of the association between registration and financing mechanisms

We found that administrative data sources are often used for indicators in social care, thus these indicators are influenced by financing structures and incentives. One of the examples given in the interviews was in a Participation law indicator. A person changing a job function within one company was registered as a job change/switch (in the data an “end of trajectory” was registered and “new beginning of trajectory” was registered). Using this data to get insight into stability in participation in society was therefore biased, and showed too many short term employer relations. According to the experts in our study, financing on population level is still more a thought than practice, however a change in the type of financing could have an effect on data availability in the social care domain. For example, there are municipalities that have started using clustered/population/performance contracts in contracting social care organisations. This means that some municipalities no longer are able to distinguish (parts of) the care provided into potentially relevant subgroups, thus registering entries under “other”, “unknown” or estimating percentages.

Improve the trust relationship between municipalities, care providers and clients

The complexity of the social care data infrastructure and processing compromises the transparency towards clients. This has consequences for the trust clients have in the processing of their data. As one expert puts it: “Unknown makes unloved.” This, together with the history of social care data uses towards control and fraud purposes has deteriorated the relationship between social care providers

For peer review only

and clients. To improve this relation a dialogue is needed between clients and social care providers to discuss the different perspectives and bring them together. Both sides need to gain the skills in order to have this dialogue effectively. Also, demonstrating what happens to the data and what the benefits this data exchange can (potential) and does (outcomes/impact) bring for the society can improve this relationship. Another approach is to change the use of data for control and fraud purposes towards citizen rights purposes. Data could be used to track down who has rights to certain social care provisions, but who is not receiving them.

Potential uses for social care data to improve the governance in integrated care practice and policies

The potential uses of social care data to improve the governance in integrated care practice and policies are broad. Experts from different perspectives stated six potential uses. 1) Cross-domain data (including social care data) can facilitate cross-domain learning and cooperation (e.g. through benchmarks), 2) Integrating social care data with population demographics, welfare, public health, and health care data can facilitate preventative measures and create early warning systems, 3) It can give insight regarding the quality and effectivity of social care in a broader perspective, 4) Better social care insights can enable a clearer accountability of social care towards contracting parties and policy, and 5) It can facilitate parties in a more data-driven governance model.

Discussion

Principle findings

This study set out to explore the landscape of social care data in the Netherlands, its current use and how it can be improved, and its current and potential use in integrated health and social care governance. We found that there is a rich array of social care data sources and information applications in the Netherlands such as public dashboards and policy monitors. Six current uses for social care data were identified: 1) communication and accountability, 2) monitoring social care policy, 3) early warning systems, 4) controls and fraud detection, 5) outreaching efforts, and 6) prioritisation. However, the present state of data and information in social care restricts it's use for integrated care governance. This can be strengthened through efforts in standardization, management of data exchange across domains, awareness of the link between registration and financing, and strengthening the overall societal trust in data sharing. Five potential uses for social care data to improve the governance in integrated care practice and policies were indicated: 1) cross-domain learning and cooperation (e.g. through benchmarks), 2) preventative measures and early warning systems, 3) give insight regarding the quality and effectivity of social care in a broader

For peer review only

perspective, 4) clearer accountability of social care towards contracting parties and policy, 5) enable cross-sector data-driven governance model.

Strengths and weaknesses of the study

This study is done in the Dutch context, however can be an inspiration to other high income countries moving from a fragmented healthcare regulated market towards regional governance of integrated care. In our desk-top research we used an explorative approach and publically available data sources. This can have biased our findings towards finding more data sources aligned with government roles. We limited our scope to data sources with a national representation which can have biased results towards finding more aggregate data. We minimized these biases by including experts from different levels (micro, meso and macro) and experts from a diverse social care data processing and use background in the interviews and reflection session to enrich the desktop study and validate results. Public sources in social care data like the dashboards and monitors used in this study are constantly developing, thus the results of the study are subject to change over time.

Possible explanations and implications on micro, meso and macro levels

Our results are characteristic for a fragmented health care system (OECD, 2022). In these systems legal and financial silos are a barrier for integrated health care, public health and social care provision governance (Bos, Klazinga, & Kringos, 2021; OECD, 2022). In the Netherlands social care responsibilities have been decentralized from the federal to the municipal level, thus differences between municipalities on data processing and use have emerged. There are recent attempts in the policy agenda to integrate care services, expressed by (among other incentives) the Integrated Care Agreement (IZA), the Healthy and Active living Agreement (GALA) and the Housing, Support and Care for the Elderly Agreement (WOZO)(Rijksoverheid, 2022a, 2022b, 2022c, 2022d). In order to implement and monitor these agreements the right information for the right person at the right time is needed in order to be actionable. But if we look at our results we see that social care data is fragmented, only standardised to a limited extent and has only suboptimal linkage possibilities with different legal and financial care silos.

A diversity of data sources have to be linked in order to appropriately monitor and be able to address health system goals such as quality, accessibility and affordability. Statistics Netherlands has shown that linkages are possible, however often only used on aggregated levels. The recent policy programmes should be accompanied by a monitoring system to monitor progress in achieving the ambitions informing the key stakeholders to support their respective roles. We found that the social care data infrastructure cannot give the individual client an overview of their integrated health and

For peer review only

social care provision, that social care providers are unable to exchange data with other health and social care professionals, that financers in social care cannot link their administrative data to financers in the health care domain, and that integrated agreements made between parties cannot be monitored using the current data infrastructure available. Recently the Dutch government has been working on an overall strategy and legislation (e.g. WEGIZ) on the information infrastructure in health and social care. But the emphasis of the approach is put on the curative health care sector. A governance structure and guidelines are needed to facilitate implementing actionable linkages across-domains and across legal and financial silos.

Future research

Our research design elicited first-hand insights into the use of social care data, filling this knowledge gap in the published literature. Policy ambitions to integrate health and social care requires adequate data and information. We suggest research on what data and information (exchanges) are needed in order to support integrated health and social care policy goals. The five potential uses found for social care data to improve the governance of integrated care practice and policies could be a focus for further research that supports implementing the desired policies.

Conclusion

This study found six current uses for social care data: 1) communication and accountability, 2) monitoring social care policy, 3) early warning systems, 4) controls and fraud detection, 5) outreaching efforts, and 6) prioritisation. Further optimization should be sought through 4 directions: standardization, management of data exchange across domains, awareness of the link between registration and financing, strengthening the overall societal trust in data sharing. The study found five potential uses for social care data to improve the governance of integrated care: 1) cross-domain learning and cooperation (e.g. through benchmarks), 2) preventative measures and early warning systems, 3) give insight regarding the quality and effectivity of social care in a broader perspective, 4) clearer accountability of social care towards contracting parties and policy, 5) enable cross-sector data-driven governance model.

Acknowledgements and PPI statement

The authors thank all citizen representatives, health and social care professionals and data experts who were willing to reflect on social data use in the Netherlands so openly during the interviews and

For peer review only

the reflection session. Patients or the public were not involved in the design, or conduct, or reporting, or dissemination plans of our research.

Funding statement

This work was supported by the European Union Funded Marie Sklodowska-Curie Innovative Training Network "HealthPros" grant number 765141.

Ethics approval and competing interests statement

This research proposal has been reviewed and approved by the Medical Ethics Review Committee of the Academic Medical Center prior to data collection (reference number: W22_136 # 22.179). No competing interests are declared.

Author's contributions

This research was drafted by VB, DK and NK. The desktop research and expert interviews were executed by VB in close collaboration and supervision of DK and NK. The reflection session was moderated by NK and accompanied by DK and VB who took notes. All authors have read and approved this manuscript.

References

Bos, V., Klazinga, N. S., & Kringos, D. S. (2021). Improving performance intelligence for governing an integrated health and social care delivery network: a case study on the Amsterdam Noord district. *BMC Health Serv Res, 21*(1), 517. doi:10.1186/s12913-021-06558-2

Centre, J. R., Studies, I. f. P. T., Abadie, F., Codagnone, C., Lupiañez-Villanueva, F., Sachinopoulou, A., & Theben, A. (2015). *Strategic intelligence monitor on personal health systems, phase 3 (SIMPHS3) : Oulu self-care (Finland), case study report*: Publications Office.

Colombo, F., Oderkirk, J., & Slawomirski, L. (2020). Health Information Systems, Electronic Medical Records, and Big Data in Global Healthcare: Progress and Challenges in OECD Countries. *Handbook of Global Health*, 1-31.

de Oliveira, C., Gatov, E., Rosella, L., Chen, S., Strauss, R., Azimaee, M., . . . Guttmann, A. (2022). Describing the linkage between administrative social assistance and health care databases in Ontario, Canada. *Int J Popul Data Sci, 7*(1), 1689. doi:10.23889/ijpds.v6i1.1689

Dixon, S. (2015). *The Employment and income effects of eight chronic and acute health conditions*: New Zealand Treasury Working Paper.

Kärki, J., & Ailio, E. (2014). The data model for social welfare in Finland. *Stud Health Technol Inform, 205*, 343-347.

Kringos, D., Carinci, F., Barbazza, E., Bos, V., Gilmore, K., Groene, O., . . . Johnsen, S. (2020). Managing COVID-19 within and across health systems: why we need performance intelligence to coordinate a global response. *Health Research Policy and Systems, 18*(1), 1-8.

McLeod, K. B., C. Tumen, S. Crichton, S. . (2015). *Using Integrated Administrative Data to Identify Youth who are at riskk of poort outcomes as adults.* . Wellington: New Zealand Treasury

For peer review only

Milne, B. J., Atkinson, J., Blakely, T., Day, H., Douwes, J., Gibb, S., . . . Teng, A. (2019). Data Resource Profile: The New Zealand Integrated Data Infrastructure (IDI). *Int J Epidemiol, 48*(3), 677-677e. doi:10.1093/ije/dyz014

OECD. (2019). *Health in the 21st Century*.

OECD. (2020a). *Realising the Potential of Primary Health Care*.

OECD. (2020b). *Who Cares? Attracting and Retaining Care Workers for the Elderly*.

OECD. (2021). *Health at a Glance 2021*.

Partners, P. (2022). Verkenning naar domeinoverstijgende samenwerkingsverbanden levert waardevolle inzichten op. In ZonMw (Ed.).

Rendle, K. A., Abramson, C. M., Garrett, S. B., Halley, M. C., & Dohan, D. (2019). Beyond exploratory: a tailored framework for designing and assessing qualitative health research. *BMJ open, 9*(8), e030123. doi:10.1136/bmjopen-2019-030123

Rijksoverheid. (2022a). *Gezond en Actief Leven Akkoord*. Den Haag Retrieved from https://www.rijksoverheid.nl/documenten/rapporten/2023/01/31/gala-gezond-en-actief-leven-akkoord

Rijksoverheid. (2022b). *Integraal Zorg Akkoord*. Den Haag Retrieved from https://www.rijksoverheid.nl/documenten/rapporten/2022/09/16/integraal-zorgakkoord-samen-werken-aan-gezonde-zorg

Rijksoverheid. (2022c). *Programma Toekomstbestendige Arbeidsmarkt Zorg en Welzijn*. Den Haag Retrieved from https://www.rijksoverheid.nl/documenten/publicaties/2022/09/30/programmatoekomstbestendigearbeidsmarktzorgwelzijn

Rijksoverheid. (2022d). *WOZO Programma Wonen, Ondersteuning en Zorg voor Ouderen*. Den Haag Retrieved from https://www.rijksoverheid.nl/documenten/rapporten/2022/11/23/programma-wonen-en-zorg-voor-ouderen

Taylor, L. A., Tan, A. X., Coyle, C. E., Ndumele, C., Rogan, E., Canavan, M., . . . Bradley, E. H. (2016). Leveraging the Social Determinants of Health: What Works? *PLoS One, 11*(8), e0160217. doi:10.1371/journal.pone.0160217

Van Driesten, G. B., S. Zonderland, S. Kraaijeveld, K. . (2021). *Zó werkt het sociaal domein* (H. Bovekerk Ed.): De Argumentenfabriek.

Wastell, D., & White, S. (2014). Making sense of complex electronic records: Socio-technical design in social care. *Applied ergonomics, 45*(2), 143-149.

Witham, M. D., Frost, H., McMurdo, M., Donnan, P. T., & McGilchrist, M. (2015). Construction of a linked health and social care database resource--lessons on process, content and culture. *Inform Health Soc Care, 40*(3), 229-239. doi:10.3109/17538157.2014.892491

World Health Organization. (2010). *Monitoring the building blocks of health systems: a handbook of indicators and their measurement strategies*: World Health Organization.

World Health Organization. (2020). *Strengthening and adjustingpublic health measures throughout the COVID-19 transition phases: policyconsiderations for the WHO European Region, 24 April 2020*. Retrieved from

Zhao, J., Gibb, S., Jackson, R., Mehta, S., & Exeter, D. J. (2018). Constructing whole of population cohorts for health and social research using the New Zealand Integrated Data Infrastructure. *Aust N Z J Public Health, 42*(4), 382-388. doi:10.1111/1753-6405.12781

For peer review only

Manuscript – Social care data and its fitness for integrated health and social care service governance: an exploratory qualitative analysis in the Dutch context

Véronique LLC Bos[1,2], Niek S Klazinga[1,2] and Dionne S Kringos[1,2]

[1] Department of Public and Occupational Health, Amsterdam UMC Location University of Amsterdam, Meibergdreef 9, Amsterdam, The Netherlands.

[2] Quality of Care, Amsterdam Public Health research institute, Amsterdam, The Netherlands.

Corresponding author

Véronique Bos

Department of Public and Occupational Health, Amsterdam UMC, University of Amsterdam

Van der Boechorststraat 7, 1081 BT Amsterdam, the Netherlands

Email: v.l.bos@amsterdamumc.nl

Supplemental Material 1 COREQ 32-item checklist

Domain 1: Research team and reflexivity

Personal Characteristics

Nr	Item	Guide question/description	
1	Interviewer/facilitator	Which author/s conducted the interview or focus group?	Interviews: VB<br><br>Reflection session: NK, DK and VB
2	Credentials	What were the researcher's credentials? E.g. PhD, MD	VB: BSc, MA<br><br>NK: Full Professor, MD<br><br>DK: Associate Professor
3	Occupation	What was their occupation at the time of the study?	VB: PhD candidate<br><br>NK: Professor of Social Medicine<br><br>DK: Associate Professor and Principal Investigator & Educator
4	Gender	Was the researcher male or female?	VB: female

For peer review only

			NK: male<br><br>DK: female
5	Experience and training	What experience or training did the researcher have?	Experienced in interviews, reflection sessions and qualitative research

Relationship with participants

Nr	Item	Guide question/description	
6	Relationship established	Was a relationship established prior to study commencement?	Before the start of the study, the study and the research team were introduced to the interviewees and reflection session experts via email.
7	Participant knowledge of the interviewer	What did the participants know about the researcher? e.g. personal goals, reasons for doing the research	In the communication to participants the background of the interviewer was listed, as well as research goals.
8	Interviewer characteristics	What characteristics were reported about the interviewer/facilitator? e.g. Bias, assumptions, reasons and interests in the research topic	The credentials, occupation and department of the research team was communicated.

Domain 2: study design

Theoretical framework

Nr	Item	Guide question/description	
9	Methodological orientation and Theory	What methodological orientation was stated to underpin the study? e.g. grounded theory,<br><br>discourse analysis, ethnography, phenomenology, content analysis	A constructivism explorative approach with inductive reasoning was used.

Participant selection

Nr	Item	Guide question/description	

For peer review only

10	Sampling	How were participants selected? e.g. purposive, convenience, consecutive, snowball	Purposive sampling based on the desktop research and participant referral
11	Method of approach	How were participants approached? e.g. face-to-face, telephone, mail, email	The participants were approached via email, phone and LinkedIn.
12	Sample size	How many participants were in the study?	18 participants in the interviews, and 10 participants in the reflection session.
13	Non-participation	How many people refused to participate or dropped out? Reasons?	3 participants dropped out without stating a reason, 1 participant refused to participate due to prioritisation issues at that moment

For peer review only

Setting

Nr	Item	Guide question/description	
14	Setting of data collection	Where was the data collected? e.g. home, clinic, workplace	Most interviews were conducted digitally or in the work environment of the interviewee. The reflection session was held digitally.
15	Presence of non-participants	Was anyone else present besides the participants and researchers?	no
16	Description of sample	What are the important characteristics of the sample? e.g. demographic data, date	Participants from different levels of the health care system (micro, meso, macro) and different perspectives (clients, providers, data processors, policy advisors) were included in the sample.

Data collection

Nr	Item	Guide question/description	

17	Interview guide	Were questions, prompts, guides provided by the authors? Was it pilot tested?	The interview guideline was grounded in the Health care performance intelligence pyramid and is provided in appendix 3. It was not pilot tested.
18	Repeat interviews	Were repeat interviews carried out? If yes, how many?	No, however some questions that could not be answered during interviews were answered at a later moment in time or corrected in the transcript by the interviewee.
19	Audio/visual recording	Did the research use audio or visual recording to collect the data?	Interviews and the reflection session were audio recorded
20	Field notes	Were field notes made during and/or after the interview or focus group?	Notes were taken by two researchers (VB and DK) during the reflection session.
21	Duration	What was the duration of the interviews or focus group?	The duration of the interviews were between 33-60 minutes.
22	Data saturation	Was data saturation discussed?	Participants could adjust content to their transcript without limitations and the reflection had the function to validate and if needed complement draft findings.
23	Transcripts returned	Were transcripts returned to participants for comment and/or correction?	Transcripts were emailed to the participants and they were given at least two weeks to review and adjust content to their transcript without limitations.

Domain 3: analysis and findings

Data analysis

Nr	Item	Guide question/description	
24	Number of data coders	How many data coders coded the data?	One researcher VB drafted the results and the reflection session

For peer review only

			was used to complement and validate principle findings.
25	Description of the coding tree	Did authors provide a description of the coding tree?	No, a reflection session with experts was used to validate principle findings.
26	Derivation of themes	Were themes identified in advance or derived from the data?	The transcript pieces were ordered by research question and themes were identified from these grouped transcript pieces.
27	Software	What software, if applicable, was used to manage the data?	Excel was used to order and theme the transcript pieces.
28	Participant checking	Did participants provide feedback on the findings?	Yes, the reflection session was used to complement and validate draft findings.

Reporting

Nr	Item	Guide question/description	
29	Quotations presented	Were participant quotations presented to illustrate the themes / findings? Was each quotation identified? e.g. participant number	One quote was used anonymously in the article and can be retraced by the researchers via the transcript identification number.
30	Data and findings consistent	Was there consistency between the data presented and the findings?	Yes, no contradicting statements were made in the interviews.
31	Clarity of major themes	Were major themes clearly presented in the findings?	There was overall consensus in the reflection session on the final principal findings.
32	Clarity of minor themes	Is there a description of diverse cases or discussion of minor themes?	In the reflection session examples were added to some statements to highlight nuances.

For peer review only

Manuscript – Social care data and its fitness for integrated health and social care service governance: an exploratory qualitative analysis in the Dutch context

Véronique LLC Bos[1,2], Niek S Klazinga[1,2] and Dionne S Kringos[1,2]

[1] Department of Public and Occupational Health, Amsterdam UMC Location University of Amsterdam, Meibergdreef 9, Amsterdam, The Netherlands.

[2] Quality of Care, Amsterdam Public Health research institute, Amsterdam, The Netherlands.

Corresponding author

Véronique Bos

Department of Public and Occupational Health, Amsterdam UMC, University of Amsterdam

Van der Boechorststraat 7, 1081 BT Amsterdam, the Netherlands

Email: v.l.bos@amsterdamumc.nl

For peer review only

Supplemental Material 2: Dashboards desktop research & Data sources

Dashboards

Waar staat je gemeente (www.waarstaatjegemeente.nl)

Gemeentezorgspiegel (https://www.vektis.nl/gemeentezorgspiegel)

Regiobeeld (https://www.regiobeeld.nl/)

Labour Market Dashboards (https://www.werk.nl/arbeidsmarktinformatie/dashboards)

Early warning dashboard for possible debt (https://monitorvroegsignaleringshv.nl)

Data sources of included indicators

CBS - Arbeidsdeelname

CBS - Bevolkingsstatistiek

CBS - Bijstandsuitkeringenstatistiek

CBS - Enquête beroepsbevolking

CBS - Enquête sociale samenhang en Welzijn

CBS - Jeugdmonitor

CBS - Gezondheidsmonitor Volwassenen en Ouderen

CBS - Gemeentelijke Monitor Sociaal Domein

CBS - Monitor Abonnementstarief

CBS – Statistiek Re-integratie door Gemeenten

Divosa - Monitor Schuldhulpverlening

Divosa - Monitor Vroegsignalering Schulden

GGD-GHOR

RIVM

Vektis

For peer review only

Manuscript – Social care data and its fitness for integrated health and social care service governance: an exploratory qualitative analysis in the Dutch context

Véronique LLC Bos[1,2], Niek S Klazinga[1,2] and Dionne S Kringos[1,2]

[1] Department of Public and Occupational Health, Amsterdam UMC Location University of Amsterdam, Meibergdreef 9, Amsterdam, The Netherlands.

[2] Quality of Care, Amsterdam Public Health research institute, Amsterdam, The Netherlands.

Corresponding author

Véronique Bos

Department of Public and Occupational Health, Amsterdam UMC, University of Amsterdam

Van der Boechorststraat 7, 1081 BT Amsterdam, the Netherlands

Email: v.l.bos@amsterdamumc.nl

Supplemental Material 3 Translated interview guide

Thank you for your time

Going through informed consent form – when agreed, proceed

Affirm the scope of the research: Wet Maatschappelijke Ondersteuning, Participatiewet, Wet Gemeentelijke Schuldhulpverlening

- What data do you collect?
  - What is the purpose of your data collection?
  - Is there underlying legislation, standardization or other agreements or confinements as to how to manage the data?
- Data input points (potential legislation/standardization at entry point)
  - Who does the entry of the data?
  - With what purpose is the data entered in the system?
  - Is there underlying legislation, standardization or other agreements or confinements as to how to enter the data?
- Data sources and custodians
  - What database is the data collected in and who is owner of the database?
- Interoperability of sources

For peer review only

   - Does the database interact with other databases? If yes, with which other databases?
- Data management responsibilities, stakeholder inclusion, and regulators
   - How is data quality managed?
   - Are stakeholders included in quality management, transferring data to performance indicators, or other ways of data management?
   - What internal/external controls are executed for the database?
- Data outputs (information) and its users
   - What indicators are presented from the database?
   - Who uses the indicators to inform their decision making?
- In your perspective how can we make good use of social care data within the three laws mentioned for the benefit of integrated care? And what are potential hurdles to make use of this data?

For peer review only

BMJ Open

Social care data and its fitness for integrated health and social care service governance: an exploratory qualitative analysis in the Dutch context

Journal:	*BMJ Open*
Manuscript ID	bmjopen-2023-078390.R1
Article Type:	Original research
Date Submitted by the Author:	06-Dec-2023

Complete List of Authors:	Bos, Véronique; Amsterdam UMC Location AMC, Department of Public and Occupational Health; Amsterdam Public Health Research Institute, Quality of Care<br>Klazinga, Niek; Amsterdam UMC Locatie AMC, Department of Public and Occupational Health; Amsterdam Public Health Research Institute, Quality of Care<br>Kringos, D; Amsterdam UMC Location AMC, Department of Public and Occupational Health; Amsterdam Public Health Research Institute, Quality of Care

**Primary Subject Heading**:	Health policy
Secondary Subject Heading:	Health informatics, Public health, Qualitative research, Evidence based practice
Keywords:	Information management < BIOTECHNOLOGY & BIOINFORMATICS, Health policy < HEALTH SERVICES ADMINISTRATION & MANAGEMENT, Organisation of health services < HEALTH SERVICES ADMINISTRATION & MANAGEMENT, Information Extraction < Information Storage and Retrieval, QUALITATIVE RESEARCH, SOCIAL MEDICINE

SCHOLARONE™
Manuscripts

I, the Submitting Author has the right to grant and does grant on behalf of all authors of the Work (as defined in the below author licence), an exclusive licence and/or a non-exclusive licence for contributions from authors who are: i) UK Crown employees; ii) where BMJ has agreed a CC-BY licence shall apply, and/or iii) in accordance with the terms applicable for US Federal Government officers or employees acting as part of their official duties; on a worldwide, perpetual, irrevocable, royalty-free basis to BMJ Publishing Group Ltd ("BMJ") its licensees and where the relevant Journal is co-owned by BMJ to the co-owners of the Journal, to publish the Work in this journal and any other BMJ products and to exploit all rights, as set out in our licence.

The Submitting Author accepts and understands that any supply made under these terms is made by BMJ to the Submitting Author unless you are acting as an employee on behalf of your employer or a postgraduate student of an affiliated institution which is paying any applicable article publishing charge ("APC") for Open Access articles. Where the Submitting Author wishes to make the Work available on an Open Access basis (and intends to pay the relevant APC), the terms of reuse of such Open Access shall be governed by a Creative Commons licence – details of these licences and which Creative Commons licence will apply to this Work are set out in our licence referred to above.

Other than as permitted in any relevant BMJ Author's Self Archiving Policies, I confirm this Work has not been accepted for publication elsewhere, is not being considered for publication elsewhere and does not duplicate material already published. I confirm all authors consent to publication of this Work and authorise the granting of this licence.

For peer review only

Social care data and its fitness for integrated health and social care service governance: an exploratory qualitative analysis in the Dutch context

Véronique LLC Bos[1,2], Niek S Klazinga[1,2] and Dionne S Kringos[1,2]

[1] Department of Public and Occupational Health, Amsterdam UMC Location University of Amsterdam, Meibergdreef 9, Amsterdam, The Netherlands.

[2] Quality of Care, Amsterdam Public Health research institute, Amsterdam, The Netherlands.

Corresponding author

Véronique Bos

Department of Public and Occupational Health, Amsterdam UMC, University of Amsterdam

Van der Boechorststraat 7, 1081 BT Amsterdam, the Netherlands

Email: v.l.bos@amsterdamumc.nl

Supplemental Material

Supplemental Material 1: Research checklist: COREQ 32-item checklist

Supplemental Material 2: Sources desk top research

Supplemental Material 3: Translated interview guide

Supplemental Material 4: Interviewee list with background/expertise

For peer review only

Abstract (298 words)

INTRODUCTION: To date, little is known on how social care data could be used to inform performance-based governance to accelerate progress towards integrated health and social care.

OBJECTIVES AND DESIGN: This study aims to perform a descriptive evaluation of available social care data in the Netherlands and its fitness for integrated health and social care service governance. An exploratory mixed method qualitative study was undertaken based on desktop research (forty-one included indicators), semi-structured expert interviews (thirteen interviews including eighteen experts) and a reflection session (ten experts).

SETTING: The Netherlands; social care is care provided in accordance with the Social Support Act, the Participation Law, and the Law for Municipal debt-counselling.

RESULTS: This study found six current uses for social care data: 1) communication and accountability, 2) monitoring social care policy, 3) early warning systems, 4) controls and fraud detection, 5) outreaching efforts, and 6) prioritisation. Further optimization should be sought through: standardization, management of data exchange across domains, awareness of the link between registration and financing, strengthening the overall trust in data sharing. The study found five ways the enhanced social care data could be used to improve the governance of integrated health and social care services: 1) cross-domain learning and cooperation (e.g. through benchmarks), 2) preventative measures and early warning systems, 3) give insight regarding the quality and effectivity of social care in a broader perspective, 4) clearer accountability of social care towards contracting parties and policy, 5) enable cross-sector data-driven governance model.

CONCLUSION: Although there are several innovative initiatives for the optimization of the use of social care data in the Netherlands, the current social care data landscape and management is not yet fit to support the new policy initiatives to strengthen integrated health and social care service governance. Directions for addressing the shortcomings are provided.

Strengths and limitations

- In our desk-top research we used an explorative approach and publically available data sources. This can have biased our findings towards finding more data sources aligned with government roles.
- We limited our scope to data sources with a national representation which can have biased results towards finding more aggregate data.
- We minimized these biases by including experts from different levels (micro, meso and macro) and experts from a diverse social care data processing and use background in the interviews and reflection session to enrich the desktop study and validate results.

For peer review only

- Public sources in social care data like the dashboards and monitors used in this study are constantly developing, thus the results of the study are subject to change over time.

Keywords (3-6)

social care data, performance information, Integrated health services, the Netherlands, informed decision making, health care governance

For peer review only

Introduction (background & problem statement)

Sound and reliable health and social care information at the right place and at the right time is essential for effective decision making in health systems [1-3]. Integrated health services, described by the WHO as "health services that are managed and delivered in a way that ensures people receive a continuum of health promotion, disease prevention, diagnosis, treatment, disease management, rehabilitation and palliative care services, at the different levels and sites of care within the health system, and according to their needs throughout their life course"(WHO, 2015), has become an important focus of many health system reforms. As a consequence, performance information presented in the traditional siloes of public health data, health care data and social care data form a hindrance for effective governance across the domains of health promotion, disease prevention, diagnosis, treatment, disease management, rehabilitation and palliative care services [4, 5].

The COVID-19 pandemic has highlighted that in order to effectively monitor and govern our health systems, data from a broad range of sources using demographics, social care data, health care data, and public health data needs to be systematically integrated. This was demonstrated by the need to monitor information in coherence and including social, behavioural and economic perspectives next to epidemiologic considerations.[6]. Countries worldwide are struggling with integrating these diverse types of data to establish comprehensive performance intelligence for decision making. Despite data rich health systems, these systems are information poor, indicating an unfulfilled potential of data and digital technologies [7]. Additionally, the COVID-19 pandemic has increased the pressure on the often already overstretched health care services and national budgets globally [8]. In order to reduce this pressure policy makers are shifting their attention to health promotion and disease prevention. Strengthening social care is part of this agenda. Investments in social care (e.g. housing, income support, nutrition support, care coordination, and community outreach) can have a positive impact on the health of citizens and health care service needs and use [9].

Countries and regions worldwide are investing in an integrated health and social care data infrastructure. The main purposes of the data infrastructures differ from primary uses of data (e.g. supporting clinical practice) to facilitate a vast range of secondary data uses (e.g. research, policy, governance) on regional or national levels. For instance, Finland's Sotkanet, a national health and social care data infrastructure, has more comprehensive data available to inform their decision making from a broader welfare perspective [10]. The affiliated Oulu Self Care Service shows that an integrated data system can also function as an enabler and improve chronic care health outcomes and efficiency

For peer review only

through shared use of health and social care data among care providers [11]. New Zealand's integrated health infrastructure (IDI) illustrates that cross-cutting data can give insights needed to govern effectively throughout the health and welfare system [12, 13]. This national integrated health infrastructure was for example used for descriptive research to characterize adolescents who experience poor health outcomes [14]. It was also used to define cohorts in the existing population; one example was a cohort of people with chronic diseases to define effects on work and income [15]. Thus, linking social care data to health care data shows potential for integrated governance, but there are also known pitfalls. A Canadian study linking administrative social assistance data to health care data warns for potential biases due to linkage errors [16]. A study in Dundee, Scotland found that linkage between health and social care data faced challenges around data linkage (e.g. use of a shared identifier across organisations), data analysis (e.g. understanding missing data), and the need for tacit knowledge (e.g. people understanding the data, its limitations and what it represents) limiting the full exploitation [17]. The Integrated Children's System in the UK initially followed a top-down managerial approach emphasizing on accountability of institutional risk instead of user-centeredness which had a detrimental effect on professional autonomy [18]. These examples show that a thorough understanding of social care data, its characteristics and use are needed to exploit its potential to gain actionable insights.

In the Netherlands multi-stakeholder policy agreements on strategic directions are considered a classical tool for governing the health- and welfare sectors. The latest policy changes aim to transform an illness insurance system into a health insurance system. In 2022, Dutch stakeholders in the health and welfare sectors launched multiple integrated policy agreements and programmes to facilitate a more integrate delivery of health and social care services: the Integrated Care Agreement (IZA), the Healthy and Active living Agreement (GALA), the Housing, Support and Care for the Elderly Programme (WOZO) and the Future-proof Care and Welfare Labour Market Programme [19-22]. In these initiatives, cooperation and integration of health and social care services and prevention efforts are highlighted as important ways forward to subdue the pressure on the healthcare system and its staff. The Netherlands has many (N: > 450) care partnerships [23]. These are often regional initiatives and alliances that are strengthening their provision of integrated health and care services for better outcomes [24]. The bandwidth of data-driven action in these collaborations is broad. Some collaborations see it as a need to have, others as a nice to have [23]. In 2015, the decentralisation of social care responsibilities increased the municipal responsibilities towards vulnerable individuals and populations. Efforts in integrated care are particularly beneficial for vulnerable individuals and populations[19-21, 25, 26]. With a high political and regional interest in integrating health and social

For peer review only

services, the Netherlands is a relevant country for an explorative study on social care data and its fitness for integrated health and social care service governance.

General aims and objectives

This study aims to perform a descriptive evaluation of the landscape of social care data in the Netherlands, its current use and how it can be improved. It attempts to explore the fitness for purpose and use of social care data to contribute to integrated health and social care service governance.

Research questions

1. What are the current uses of social care data in the Netherlands, and how can the usability of this data be enhanced?
2. In what ways can enhanced social care data contribute to the governance of integrated health and social care services?

Research Methods

Research design and scope

A constructivism exploratory qualitative study design [27] was employed using: 1) desktop research, 2) semi-structured expert interviews and 3) a reflection session. The study conforms to the Consolidated Criteria for Reporting Qualitative Studies, the 32-item checklist for this study can be found in Supplemental Material 1. Our research design was grounded in the Health care performance intelligence pyramid [1]. The hierarchical pyramid defines how measurement through data collection and its translation to indicators is at the base of governance and management through information and knowledge. Action and utilisation is the ultimate end goal and needs its own translation from information and knowledge to practice. Each of these steps needs to consider the regulatory, organisational, political and cultural context.

The scope of this research is social care as provided within the municipal responsibilities in the Netherlands through: 1) the Social Support Act (Wet Maatschappelijke Ondersteuning, WMO); regulating municipal responsibility for supplementing citizens in their care need, 2) the Participation Law (Participatiewet); regulating municipal responsibility for supporting citizens in participating in the society, and 3) the Law for Municipal debt-counselling (Wet Gemeentelijke Schuldhulpverlening); regulating municipal responsibility for supporting citizens in financial need. As the focus of this study is on the adult population we have excluded social care as provided by the Youth Act (Jeugdwet);

For peer review only

regulating the preventative and mental health care for children. As social care was decentralised to municipal level in 2015 in the Netherlands, we have only included documents from beyond this time point in our desktop research.

Patient and Public Involvement

Clients, patients and the public have not been involved in the design or conduct of the study. Social care clients were represented in the interviews and the reflection session by the National (social care) Client Council. The results of the study will be disseminated through our network and shared with all study participants.

Data collection and coding

Our data collection took place from January 2022 to October 2022. The desktop research aimed to get an overview of publicly available social care indicators. We used two starting points: 1) the website vzinfo.nl of the National Institute for Public Health and the Environment commissioned by the Ministry of Health, Welfare and Sport, which provides an overview and metadata of available public health and care information and 2) a social care stakeholder analysis done by Driesten and Blink in 2021 [28]. Data included in this study was either collected on a national level, or purposefully sampled to represent the national population. Data(sets) that were collected only for a single organisation/municipality or region were excluded from this study. We collected all described indicators from dashboards and reports found via these two starting points that met the scope of this research. An overview of all the collected dashboards and reports included in the desktop research and their references can be found in Supplemental Material 2.

The expert interviews aimed to complement the desktop research and highlight current uses of social care data and indicators within the social care domain, to describe ways to optimize social care data (infrastructure), and to highlight potential uses for integrated health and social care service governance. We purposively sampled experts based on the sources found in the desktop research, and gave them the opportunity to refer to other experts in the field of social care data in the Netherlands. The participants were approached via email, phone and LinkedIn. We aimed to have at least two representatives from each level of the health care system: micro (social care clients and care professionals), meso (data custodians and data processing organizations), and macro (policy and legal experts) levels. Most interviews were organized digitally in the aftermath of the COVID-19 pandemic and the duration of the interviews was between thirty-three and sixty minutes. Three approached participants dropped out without stating a reason, one approached participant could not to participate due to prioritisation issues at that moment. The interview guideline (Supplemental Material 3) was

For peer review only

grounded in the Health care performance intelligence pyramid. Before the start of the study, research goals and the research team (department, credentials and occupation) were introduced to the interviewees via email. With the written informed consent of participants, all interviews were audio-recorded and transcribed. Transcripts were emailed to the participants and they were given at least two weeks to review and adjust the content of their transcript without limitations. The validated transcripts were used for the thematic analysis.

The reflection session was organized on 12 October 2022 with the following aims: 1) to validate the draft results from the desktop research and semi-structured interviews and 2) to consolidate key findings. It was organized digitally in the aftermath of the COVID-19 pandemic. First, the study team presented the draft results. Second, participants shared their general reflections on the draft results. Finally, the following questions were discussed in-depth: 1) Is the outlined landscape of available social care data and indicators, its management and its use recognisable?, 2) How can we optimize the use of available social care data within the social care domain?, 3) How can social care data contribute to public health and health care? And vice versa?, and 4) How can social care data be used in governing towards regional integrated care provision? The reflection session was audio recorded with the verbal informed consent of all participants. The draft results from the desktop study and expert interviews were shared with the participants in advance.

Data analysis

To answer research question one, we used information retrieved from desktop research and interviews. First, we constructed an overview of all dashboards and documents collected with their data custodian and a qualitative description of their content (Supplemental Material 2). Then we abstracted all indicators within the scope of this research and grouped them in their legal silos. Finally, we categorized them into the following subgenres: input, process and output, outcome, and impact and allocated source types (administrative, medical record, registry, survey, direct observation) to each indicator. Transcript pieces that described available social care data in the Netherlands were used to enrich the desktop research by adding additional data sources. We selected and then grouped all pieces of the transcripts from the interviews that described social care data in the Netherlands, its current use and how it can be improved. Then we analysed the transcript pieces using an analytic induction process using key words and statements to define how social care data is currently used and can be improved.

To answer research question two, pieces of the interview transcripts were grouped that relate to the current and potential use of social care data to improve integrated health and social care service

For peer review only

governance. Then an analytic induction process using key words and statements was used to define how social care data was used for integrated health and social care service governance. The draft results of research question one and two were presented, enriched, clarified and verified in the reflection session.

Results

Characteristics of the data sources and informants

The sources included in the desktop research can be found in Supplemental Material 2. Thirteen interviews were conducted with a total of eighteen experts including representatives for social care clients and providers , representatives from the municipalities, social care administrative data processors, owners of social care dashboards and indicator reports and experts from the health care insurance data field with experience in integrated care projects. Three participants made minor changes to their interview transcript. The changes contained clarifications of the content and additions to the content. No changes to the transcripts were made that affected the results. The interviewee list with background/expertise can be found in Supplemental Material 4. The reflection session included ten experts on social care data, including representatives for social care clients (n=1) and providers (n=2), representatives from the municipalities (n=2), social care administrative data processors (n=4, from 3 different organizations and working with different social care data), and an expert from the health care insurance data field with experience in integrated care projects (n=1).

What social care data is available in the Netherlands and how is it used?

Measuring social care data: data collection and Indicators

The most common data source types used to populate the included social care indicators were administrative data (including the needs assessment done for administrative purposes) and surveys (often samples representative to national population). Indicators on use and costs of services were most common and most often reported on municipal or neighbourhood level. These indicators were often updated once or twice a year. There is a diversity in measurement instruments to measure an individual’s needs for social care. However, there is a national guideline for a needs assessment obligatory in order to receive the social care provided. Prediction models (e.g. the Social Support Act prediction model) are available. However, one expert mentioned that policymakers find such models difficult to apply due to the lack of data driven personnel and a data driven work culture. Policy makers are also hesitant to use these models as they are by definition incorrect, as they only model reality and can prove to be incorrect in hindsight.

For peer review only

The use of client reported data for decision making is very limited in the social care domain. There is a legal obligation in the Social Support Act for municipalities to carry out a national client experiences measure. Until 2021 this measure consisted of 10 nationally defined questions divided in three themes: 1) access to services, 2) quality of the services provided, and 3) the effect on the independence for the client and his/her participation in society. However, since 2021, due to suboptimal use of the measure, municipalities are free to adapt the client experience measure to their needs, either using the 10 defined questions, adding additional questions or using their own survey format. Experts state that this tailoring of the survey by municipalities might be detrimental to cross-municipality comparisons. Besides the national client experience measure, client stories are occasionally used to represent clients' voices to complement the data provided, this is relatively customary in the municipal reporting on social care.

The tables below give an overview of all included indicators and their defined indicator and data source type per legal silo.

For the Social Support Act we included 17 indicators (table 1). Most were sourced from survey data or administrative data and gave insight on (potential) inputs or processes and outputs.

Table 1 Social care indicators included in the desktop study within scope of the Social Support Act

Indicator	Type of indicator	Type of source
Trend in the number of potential Informal carers	(potential) Input	survey data
% Vulnerable population	(potential) Input	administrative / survey / medical record data
% of people with one or more limitation in daily functioning	(potential) Input	survey data
% of people with one or more restrictions in their mobility	(potential) Input	survey data
% of people with restrictions in their eye sight	(potential) Input	survey data
% of people with hearing disabilities	(potential) Input	survey data
Budgeted and actual costs for Social Support Act	Input	administrative
Municipal policy (only customized provisions, mainly customized provisions, as many general as customized provisions, mainly general provisions, only general provisions) of Social Support Act care provision	Input	survey data
Ratio between households with and without care provision	Process & Output	administrative
Relative number of households with provisions of care and support by number of provisions	Process & Output	administrative
Relative number of households with provisions of care and support by number of provisions per domain	Process & Output	administrative
Trend number of inhabitants with Social Support Act care provision	Process & Output	administrative

For peer review only

Relative number of inhabitants with Social Support Act care provision by category	Process & Output	administrative
Number of inhabitants with Social Support Act care provision	Process & Output	administrative
Relative number of inhabitants in sheltered housing by region	Process & Output	survey data / administrative
Number of terminated Social Support Act provisions by reason (total, deceased, planned/anticipated termination, move to other municipality, other/unknown)	Process & Output	administrative
Qualitative reporting of the WMO Client experience outcomes	Outcome	survey data

For the Participation law we included 19 indicators (table 2). All were sourced from survey data or administrative data and most gave insight on processes and outputs or outcomes.

Table 2 Social care indicators included in the desktop study within scope of the Participation law

Indicator	Type of indicator	Type of source
Trend in pension age	Input	survey data
Ratio between households with and without care provision	Process & Output	administrative
Relative number of households with provisions of care and support by number of provisions	Process & Output	administrative
Relative number of households with provisions of care and support by number of provisions per domain	Process & Output	administrative
Number of inhabitants with a welfare benefit	Process & Output	administrative
Number of inhabitants with a welfare benefit by municipality and reference group	Process & Output	administrative
Number of inhabitants with a reintegration facility	Process & Output	administrative
Relative number of inhabitants with a reintegration facility by municipality and reference group	Process & Output	administrative
Number of unemployed inhabitants in working age	Outcome	survey data
% inhabitants participating in formal work, volunteer work and informal care by sex	Outcome	survey data
% of persons with/without health problems with formal work by age categories	Outcome	survey data
% of the Formal work population with/without health problems by age categories	Outcome	survey data
% of net formal work participation by age category and sex	Outcome	survey data
Number of inhabitants of the formal workers working part-time by sex	Outcome	survey data
% Unemployment by age category and sex	Outcome	survey data
Trend % net formal work participation by two age categories and sex	Outcome	survey data
Trend % unemployment by age category and sex	Outcome	survey data
% Unemployed young professionals	Outcome	survey data
SES-WOA score (financial prosperity, level of education and recent employment history of private households)	Impact	administrative

For peer review only

For the Law for Municipal debt-counselling we included 9 indicators (table 3). All were sourced from survey data or administrative data and most gave insight on processes and outputs.

Table 3 Social care indicators included in the desktop study within scope of the Law for Municipal debt-counselling

Indicator	Type of indicator	Type of source
Municipal capacity to provide debt counselling services (enough capacity for demand, enough capacity for demand, but sustainability for the future is under pressure, not enough capacity for demand)	Input	survey data
Factors that determine the manner in which early warning signals for over indebtedness are followed up (type of notification, debt size, type of non-payment, age, composition of the household, time passed since notification, postal code)	Input	administrative / survey data
Number of applications for municipal debt counselling services	Process & Output	survey data
Qualitative reporting by cooperating municipalities on the (change in) target group for municipal debt counselling services	Process & Output	survey data
Number of early warning signals (one non-payment from one specific provider on one home address) per 1000 inhabitants per municipality	Process & Output	administrative
Number of early warning notifications (multiple signals at one address) per municipality	Process & Output	administrative
Number of contacts (defined as contact with a reaction, e.g. reply to email, opening the door, answering the phone call) resulting from early warning signals	Process & Output	administrative
% of inhabitants accepting the offer for debt counselling services	Process & Output	Administrative
SES-WOA score (financial prosperity, level of education and recent employment history of private households)	Impact	administrative

Governance and management in social care data: rules for translation to information and knowledge

National administrative registration standards are in place, for example the i-WMO standard. However, municipalities have the legal mandate for the social care provision. This mandate also includes how to structure and use (administrative) social care data. This makes national level benchmarking between municipalities difficult, due to a high variability in interpretation and use of the available national standards. The diversity of payment models within and across municipalities also impact the way municipalities structure this data, making this data only interpretable if: 1) within a known context (for example on municipal level) or 2) aggregated to very general terms

For peer review only - http://bmjopen.bmj.com/site/about/guidelines.xhtml

where this variation is diminished. Our sources also showed a big variety between municipalities in data processing power, ranging from own data processing departments to support policy decisions to the minimal data processing needed for accountability purposes. This variety in data processing power also exists between social care provider organizations.

Legislation on the use of social care data mainly covers accountability of social provisions (fraud and supervision), the use of the citizen service number for identification, and permission required for use of personal data (privacy). Multiple public and private parties can fulfil the role of data processor in social care in the Netherlands, however often under municipal authority. But the use of social care data for quality improvement of care provision by integrated provider networks, research or policy action is not defined in the legislation. Thus, a weighing between respecting privacy of personal data on the one hand and having data available to deliver adequate social care services or take timely policy action on the other hand, cannot be made.

Utilisation of social care data: what is done with the acquired knowledge through data

Social care data is mostly used in the *communication and accountability* between care providers and financers (e.g. official needs assessments or registration of start and end a care trajectory), and in *monitoring social care policy* on municipal and national levels (e.g. Monitor on adult health and health of the elderly). There is an *early warning system* on over-indebtedness using non-payments of housing, utilities and health care insurance to alert municipalities to a potential demand in social care services to prevent over-indebtedness. According to one of the care organisations interviewed this proactive exchange of information has led to the prompt identification of individuals requiring services, thereby minimizing the need for involuntary out-of-house placements in the serviced district. Nevertheless, additional insights from interviewed experts underscored that in numerous regions, the transition from data availability to utilizing that data for identification and subsequent action by municipalities, necessary for achieving impact, still requires improvement. Data alone does not lead to action.

Traditionally, data in the social care domain has been used for *controls and fraud detection*, which has led to a distrust in data exchange in the social care domain from the care provider and client perspective. Examples of information used for controls and fraud detection mentioned by interviewees were: 1) financial information on care providers to guide the municipal purchasing risk management strategy and 2) information on which clients have access to Long Term Care Act resources to check whether the Social Support Act resources given are still relevant. Some recent examples rethink this approach, like the *outreaching efforts* of the Social Insurance Bank (SVB) to track and locate eligible citizens entitled to receive a pension, yet for reasons unknown, have not

For peer review only

(yet) applied for it. We've identified an illustrative *prioritisation* case where a social work organization employed national demographic data in tandem with their client base to evaluate whether they are effectively directing their efforts toward the neighbourhood exhibiting the highest (predicted) need for debt counselling.

Improving the social care data use

Data: Standardization of registration

A high variation between municipalities in social care data, data infrastructure and processing (power) is the result of policy decentralization efforts aiming to provide care 'closer to home'. Efforts to standardize product codes have already yielded results (from 100.000 product codes in 2015 to about 3.000-4.000 product codes per law during the time of this research). However, some experts interviewed believe that more standardization in social care data, its infrastructure and its processing can increase comparability and decrease administrative burden on the long run for care provider organizations and municipalities alike. We found heterogeneous roles and responsibilities in the coordination and alignment of data processing in social care. It has become difficult to compare even similar indicators due to source and processing variability. Establishing a well-defined governance structure for social care data, along with a robust infrastructure and processing framework, could enhance the stability and interpretability of the available data for more effective utilization. Standardization initiatives can provide a coherent framework for data registration, thereby mitigating interpretation challenges associated with the registration process. Representatives from social care organizations emphasize the importance of considering that standardizing data registration does not imply a necessity to diminish regional variability in care delivery, especially when diverse regions exhibit distinct care needs.

Governance and management of social care data across domains and within clear roles for a societal purpose

Data processing is often operationalized per legal and financial silo. Experts state that in order to have a clear picture of the societal needs and impact of social care, insight in performance information across legal and financial silos is needed (e.g. the association between over-indebtedness and health care use). However, data use across domains is legally and politically restricted in the Netherlands. Additionally, cross-sector data sharing, including across private and public interests, may have more potential for *preventative measures*, but is politically sensitive. For

For peer review only

example, in the context of nationally organized debt counselling initiatives, it is noteworthy that study loans exhibit a higher average credit per person compared to consumer credits. However, it's essential to acknowledge that study loans, in contrast to consumer credits, face less transparent monitoring by credit providers, primarily due to political sensitivity. The implementation of these supplementary prevention options could yield positive effects, provided they operate within the requisite boundaries of a robust ethical and legal framework. This ensures alignment between data use and its intended societal purpose while avoiding any potential discrepancies.

Multiple experts state that egislation concerning data exchange and processing ought to prioritize explicit use and purpose rather than merely restricting (while simultaneously facilitating extensive) data processing and integration within specific financial or legal silos. The measurement of service effectiveness and affordability remains constrained, as does the availability of metrics aligned with newly articulated national policy goals, such as the Integrated Care Agreement. Currently, there is limited data on the well-being of citizens, including how to sustain healthy lives and identify potential risks to health.

Awareness of the association between registration and financing mechanisms

We found that indicators in social care frequently rely on administrative data sources, leading to a notable influence from financing structures and incentives on these indicators. One of the examples given in the interviews was in a Participation law indicator. When an individual transitions to a different role within the same company, the data records this as a job change or switch, with an "end of trajectory" and a subsequent "new beginning of trajectory" being registered. Leveraging this data to gain insights into stability in societal participation was inherently biased, revealing an overemphasis on short-term employer relations.

According to the experts in our study, financing on a population level remains more theoretical than practical. Nonetheless, a shift in financing models could potentially impact data availability within the social care domain. For example, there are municipalities that have started using clustered/population/performance contracts in contracting social care organisations. This means that some municipalities no longer are able to distinguish (parts of) the care provided into potentially relevant subgroups, thus registering entries under “other”, “unknown” or estimating percentages.

Improve the trust relationship between municipalities, care providers and clients

The complexity of the social care data infrastructure and processing compromises the transparency towards clients. This has consequences for the trust clients have in the processing of their data. As one expert puts it: “Unknown makes unloved.” This, together with the history of social care data uses

For peer review only

towards control and fraud purposes has deteriorated the relationship between social care financers (e.g. municipalities) and clients. To improve this relation a dialogue is needed between clients and social care financers to discuss the different perspectives and bring them together. Both parties must acquire the necessary skills to engage in this dialogue effectively. Additionally, illustrating the processes involved in data exchange and showcasing the potential benefits, as well as the actual outcomes and impact it brings to society, can enhance this relationship. An alternative approach involves shifting the utilization of data from control and fraud detection purposes to prioritizing citizen rights. Data could then be employed to identify individuals entitled to specific social care provisions but are not currently receiving them.

Potential uses for social care data to improve the governance in integrated care practice and policies

Experts state that the enhanced social care data can contribute to the governance of integrated health and social care services . Five potential uses were stated: 1) Cross-domain data (including social care data) can facilitate cross-domain learning and cooperation (e.g. through benchmarks), 2) Integrating social care data with population demographics, welfare, public health, and health care data can facilitate preventative measures and create early warning systems, 3) It can give insight regarding the quality and effectivity of social care in a broader perspective, 4) Better social care insights can enable a clearer accountability of social care towards contracting parties and policy, and 5) It can facilitate parties in a more data-driven governance model.

Discussion

Principle findings

This study aimed to investigate the current applications of social care data in the Netherlands, identify areas for improvement, and assess how enhanced social care data can contribute to the governance of integrated health and social care services. We found that there is a rich array of social care data sources and information applications in the Netherlands such as public dashboards and policy monitors. Six current uses for social care data were identified: 1) communication and accountability, 2) monitoring social care policy, 3) early warning systems, 4) controls and fraud detection, 5) outreaching efforts, and 6) prioritisation. However, the present state of data and information in social care restricts its use for integrated care governance. Enhancing the data involves initiatives in standardization, effective management of data exchange across domains, recognizing the connection between registration and financing, and bolstering societal trust in data sharing overall. Five potential ways were identified to use this enhanced data to improve the

For peer review only

governance in integrated health and social care services: 1) cross-domain learning and cooperation (e.g. through benchmarks), 2) preventative measures and early warning systems, 3) give insight regarding the quality and effectivity of social care in a broader perspective, 4) clearer accountability of social care towards contracting parties and policy, 5) enable cross-sector data-driven governance model.

Strengths and weaknesses of the study

This study is conducted within the Dutch context but can serve as inspiration for other high-income countries transitioning from a fragmented healthcare regulated market to regional governance of integrated care. In our desk-top research we used an explorative approach and publically available data sources. This can have biased our findings towards finding more data sources aligned with government roles. We limited our scope to data sources with a national representation which can have biased results towards finding more aggregate data. We minimized these biases by including experts from different levels (micro, meso and macro) and experts from a diverse social care data processing and use background in the interviews and reflection session to enrich the desktop study and validate results. Public sources in social care data like the dashboards and monitors used in this study are constantly developing, thus the results of the study are subject to change over time. This research was designed to be a starting point and not to exhaust all options. Different stakeholder settings could generate different results. We have limited the bias by intentionally taking available public indicators as a starting point for stakeholders to reflect on and by including a wide variety of stakeholders in our interviews and the reflection session.

Possible explanations and implications on micro, meso and macro levels

Our results are characteristic for a fragmented health care system (OECD, 2022). In these systems legal and financial silos are a barrier for integrated health care, public health and social care provision governance (Bos, Klazinga, & Kringos, 2021; OECD, 2022). In the Netherlands social care responsibilities have been decentralized from the federal to the municipal level, resulting in variations between municipalities regarding data processing and utilization. There are recent attempts in the policy agenda to integrate care services, expressed by (among other incentives) the Integrated Care Agreement (IZA), the Healthy and Active living Agreement (GALA) and the Housing, Support and Care for the Elderly Agreement (WOZO)[19-22]. To effectively implement and oversee these agreements, actionable information tailored to the right individual at the right time is essential. However, our findings reveal that social care data is fragmented, standardized to a limited extent, and offers suboptimal linkage possibilities with various legal and financial care silos. Despite these challenges, there is a current trend toward increased integration, endorsed by a majority of political parties in the Netherlands. The

For peer review only

outcomes of the November 2023 elections will additionally influence political support for further reforms in financing arrangements aligned with integrated care ambitions.

To effectively monitor and address health system goals, including quality, accessibility, and affordability, it is imperative to link a diverse range of data sources. Statistics Netherlands has demonstrated the feasibility of linkages, although they are frequently employed only at aggregated levels. The recent policy programmes should be accompanied by a monitoring system to monitor progress in achieving the ambitions informing the key stakeholders to support their respective roles. Our findings indicate that the existing social care data infrastructure falls short in providing individual clients with a comprehensive overview of their integrated health and social care provisions. Additionally, social care providers face challenges in exchanging data with other health and social care professionals. Financers in social care encounter difficulties linking their administrative data to health care domain financers, and the current data infrastructure does not adequately support the monitoring of integrated agreements made between different parties. Recently the Dutch government has been working on an overall strategy and legislation (e.g. Electronic Data Sharing in Health Care Act) on the information infrastructure in health and social care[29, 30]. But the emphasis of the approach is put on the curative health care sector. Establishing a governance structure and guidelines is crucial to facilitate the implementation of actionable linkages across domains and within various legal and financial silos.

Future research

Our research design has provided first-hand insights into the utilization of social care data, addressing a notable knowledge gap in the existing published literature. Policy ambitions to integrate health and social care requires adequate data and information. We recommend further research to identify the essential data and information exchanges required to support integrated health and social care policy goals. The five potential uses found for social care data to improve the governance of integrated care practice and policies could be a focus for further research that supports implementing the desired policies.

Conclusion

This study identified six current uses for social care data: 1) communication and accountability, 2) monitoring social care policy, 3) early warning systems, 4) controls and fraud detection, 5) outreaching efforts, and 6) prioritization. Further optimization is recommended through standardization, effective

For peer review only

management of data exchange across domains, increased awareness of the link between registration and financing, and reinforcing societal trust in data sharing. Additionally, the study revealed five ways in which enhanced social care data could be employed to govern integrated health and social care services: 1) fostering cross-domain learning and cooperation (e.g., through benchmarks), 2) implementing preventative measures and early warning systems, 3) providing insight into the quality and effectiveness of social care from a broader perspective, 4) enhancing accountability of social care toward contracting parties and policy, and 5) enabling a cross-sector data-driven governance model.

Acknowledgements

The authors thank all citizen representatives, health and social care professionals and data experts who were willing to reflect on social data use in the Netherlands so openly during the interviews and the reflection session.

Contributership statement

This research was drafted by VB (Female, BSc, MA; PhD candidate), DK (Female, Associate Professor and Principal Investigator & Educator) and NK (Male, Full Professor of Social Medicine, MD). The desktop research and expert interviews were executed by VB (Experienced in interviews, reflection sessions and qualitative research) in close collaboration and supervision of DK and NK. The reflection session was moderated by NK and accompanied by DK and VB who took notes. All authors have read and approved this manuscript.

Competing interests

No competing interests are declared.

Funding

This work was supported by the European Union Funded Marie Sklodowska-Curie Innovative Training Network "HealthPros" grant number 765141.

Data sharing statement

The data that support the findings of this study are available on request from the corresponding author (VB) for checks on scientific integrity or quality control by licensed parties. For other reasons, due to the personal nature of this data, permission of the participants is required.

For peer review only

Ethics approval

This research proposal has been reviewed and approved by the Medical Ethics Review Committee of the Academic Medical Center prior to data collection (reference number: W22_136 # 22.179).

References

1. Kringos, D., et al., *Managing COVID-19 within and across health systems: why we need performance intelligence to coordinate a global response.* Health Research Policy and Systems, 2020. **18**(1): p. 1-8.
2. World Health Organization, *Monitoring the building blocks of health systems: a handbook of indicators and their measurement strategies*. 2010: World Health Organization.
3. Colombo, F., J. Oderkirk, and L. Slawomirski, *Health Information Systems, Electronic Medical Records, and Big Data in Global Healthcare: Progress and Challenges in OECD Countries.* Handbook of Global Health, 2020: p. 1-31.
4. OECD, *Who Cares? Attracting and Retaining Care Workers for the Elderly*. 2020.
5. OECD, *Realising the Potential of Primary Health Care*. 2020.
6. World Health Organization, *Strengthening and adjustingpublic health measures throughout the COVID-19 transition phases: policyconsiderations for the WHO European Region, 24 April 2020*. 2020, World Health Organization. Regional Office for Europe.
7. OECD, *Health in the 21st Century*. 2019.
8. OECD, *Health at a Glance 2021*. 2021.
9. Taylor, L.A., et al., *Leveraging the Social Determinants of Health: What Works?* PLoS One, 2016. **11**(8): p. e0160217.
10. Kärki, J. and E. Ailio, *The data model for social welfare in Finland.* Stud Health Technol Inform, 2014. **205**: p. 343-7.
11. Centre, J.R., et al., *Strategic intelligence monitor on personal health systems, phase 3 (SIMPHS3) : Oulu self-care (Finland), case study report*, ed. F. Abadie and C. Codagnone. 2015: Publications Office.
12. Milne, B.J., et al., *Data Resource Profile: The New Zealand Integrated Data Infrastructure (IDI).* Int J Epidemiol, 2019. **48**(3): p. 677-677e.
13. Zhao, J., et al., *Constructing whole of population cohorts for health and social research using the New Zealand Integrated Data Infrastructure.* Aust N Z J Public Health, 2018. **42**(4): p. 382-388.
14. McLeod, K.B., C. Tumen, S. Crichton, S. , *Using Integrated Administrative Data to Identify Youth who are at riskk of poort outcomes as adults.* . 2015, New Zealand Treasury: Wellington.
15. Dixon, S., *The Employment and income effects of eight chronic and acute health conditions*. 2015: New Zealand Treasury Working Paper.
16. de Oliveira, C., et al., *Describing the linkage between administrative social assistance and health care databases in Ontario, Canada.* Int J Popul Data Sci, 2022. **7**(1): p. 1689.
17. Witham, M.D., et al., *Construction of a linked health and social care database resource--lessons on process, content and culture.* Inform Health Soc Care, 2015. **40**(3): p. 229-39.
18. Wastell, D. and S. White, *Making sense of complex electronic records: Socio-technical design in social care.* Applied ergonomics, 2014. **45**(2): p. 143-149.
19. Rijksoverheid, *Integraal Zorg Akkoord*. 2022: Den Haag.
20. Rijksoverheid, *WOZO Programma Wonen, Ondersteuning en Zorg voor Ouderen*. 2022: Den Haag.
21. Rijksoverheid, *Gezond en Actief Leven Akkoord*. 2022: Den Haag.

For peer review only

22. Rijksoverheid, *Programma Toekomstbestendige Arbeidsmarkt Zorg en Welzijn*. 2022: Den Haag.
23. Partners, P., *Verkenning naar domeinoverstijgende samenwerkingsverbanden levert waardevolle inzichten op*, ZonMw, Editor. 2022.
24. Bos, V., N.S. Klazinga, and D.S. Kringos, *Improving performance intelligence for governing an integrated health and social care delivery network: a case study on the Amsterdam Noord district.* BMC Health Serv Res, 2021. **21**(1): p. 517.
25. Kasteridis, P., et al., *Who would most benefit from improved integrated care? Implementing an analytical strategy in South Somerset.* Int J Integr Care, 2015. **15**: p. e001.
26. Society, T.C.o.P.H., *Met de stroom mee: Naar een duurzaam en adaptief stelsel van zorg en ondersteuning*. 2023: The Hague.
27. Rendle, K.A., et al., *Beyond exploratory: a tailored framework for designing and assessing qualitative health research.* BMJ Open, 2019. **9**(8): p. e030123.
28. Van Driesten, G.B., S. Zonderland, S. Kraaijeveld, K. , *Zó werkt het sociaal domein*. 2021: De Argumentenfabriek.
29. Rijksoverheid, *Electronic Data Sharing in Health Care Act*. 2023: The Hague.
30. Ministry of Health, W.a.S., *National Strategy on the Health Information system in the Netherlands*. 2023.

For peer review only

Manuscript – Social care data and its fitness for integrated health and social care service governance: an exploratory qualitative analysis in the Dutch context

Véronique LLC Bos[1,2], Niek S Klazinga[1,2] and Dionne S Kringos[1,2]

[1] Department of Public and Occupational Health, Amsterdam UMC Location University of Amsterdam, Meibergdreef 9, Amsterdam, The Netherlands.

[2] Quality of Care, Amsterdam Public Health research institute, Amsterdam, The Netherlands.

Corresponding author

Véronique Bos

Department of Public and Occupational Health, Amsterdam UMC, University of Amsterdam

Van der Boechorststraat 7, 1081 BT Amsterdam, the Netherlands

Email: v.l.bos@amsterdamumc.nl

Supplemental Material 1 COREQ 32-item checklist

Domain 1: Research team and reflexivity

Personal Characteristics

Nr	Item	Guide question/description	Answer and where to find in manuscript
1	Interviewer/facilitator	Which author/s conducted the interview or focus group?	Interviews: VB<br><br>Reflection session: NK, DK and VB<br><br>Contributorship statement page 19
2	Credentials	What were the researcher's credentials? E.g. PhD, MD	VB: BSc, MA<br><br>NK: Full Professor, MD<br><br>DK: Associate Professor<br><br>Contributorship statement page 19
3	Occupation	What was their occupation at the time of the study?	VB: PhD candidate<br><br>NK: Professor of Social Medicine

For peer review only

			DK: Associate Professor and Principal Investigator & Educator<br><br>Contributorship statement page 19
4	Gender	Was the researcher male or female?	VB: female<br><br>NK: male<br><br>DK: female<br><br>Contributorship statement page 19
5	Experience and training	What experience or training did the researcher have?	Experienced in interviews, reflection sessions and qualitative research<br><br>Contributorship statement page 19

Relationship with participants

Nr	Item	Guide question/description	
6	Relationship established	Was a relationship established prior to study commencement?	Before the start of the study, the study and the research team were introduced to the interviewees and reflection session experts via email.<br><br>Research Methods pages 7-8
7	Participant knowledge of the interviewer	What did the participants know about the researcher? e.g. personal goals, reasons for doing the research	In the communication to participants the background of the interviewer was listed, as well as research goals.<br><br>Research Methods pages 7-8
8	Interviewer characteristics	What characteristics were reported about the interviewer/facilitator? e.g. Bias, assumptions, reasons and interests in the research topic	The credentials, occupation and department of the research team was communicated.<br><br>Research Methods pages 7-8

For peer review only

Domain 2: study design

Theoretical framework

Nr	Item	Guide question/description	
9	Methodological orientation and Theory	What methodological orientation was stated to underpin the study? e.g. grounded theory, discourse analysis, ethnography, phenomenology, content analysis	A constructivism explorative approach with inductive reasoning was used. Research Methods page 6

Participant selection

Nr	Item	Guide question/description	
10	Sampling	How were participants selected? e.g. purposive, convenience, consecutive, snowball	Purposive sampling based on the desktop research and participant referral Research Methods page 7
11	Method of approach	How were participants approached? e.g. face-to-face, telephone, mail, email	The participants were approached via email, phone and LinkedIn. Research Methods page 7
12	Sample size	How many participants were in the study?	18 participants in the interviews, and 10 participants in the reflection session. Results page 9
13	Non-participation	How many people refused to participate or dropped out? Reasons?	3 participants dropped out without stating a reason, 1 participant refused to participate due to prioritisation issues at that moment Research Methods page 7

Setting

Nr	Item	Guide question/description	
14	Setting of data collection	Where was the data collected? e.g. home, clinic, workplace	Most interviews were conducted digitally or in the work environment of the interviewee.

For peer review only

			The reflection session was held digitally.<br><br>Research Methods page 7
15	Presence of non-participants	Was anyone else present besides the participants and researchers?	No<br><br>Contributorship statement page 19
16	Description of sample	What are the important characteristics of the sample? e.g. demographic data, date	Participants from different levels of the health care system (micro, meso, macro) and different perspectives (clients, providers, data processors, policy advisors) were included in the sample.<br><br>Research Methods page 7

For peer review only

Data collection

Nr	Item	Guide question/description	
17	Interview guide	Were questions, prompts, guides provided by the authors? Was it pilot tested?	The interview guideline was grounded in the Health care performance intelligence pyramid and is provided in appendix 3. It was not pilot tested.<br><br>Research Methods page 7
18	Repeat interviews	Were repeat interviews carried out? If yes, how many?	No, however some questions that could not be answered during interviews were answered at a later moment in time or corrected in the transcript by the interviewee.<br><br>Research Methods page 8
19	Audio/visual recording	Did the research use audio or visual recording to collect the data?	Interviews and the reflection session were audio recorded<br><br>Research Methods page 8

20	Field notes	Were field notes made during and/or after the interview or focus group?	Notes were taken by two researchers (VB and DK) during the reflection session.<br><br>Contributor statement page 19
21	Duration	What was the duration of the interviews or focus group?	The duration of the interviews were between 33-60 minutes.<br><br>Research Methods page 7
22	Data saturation	Was data saturation discussed?	Participants could adjust content to their transcript without limitations and the reflection had the function to validate and if needed complement draft findings.<br><br>Research Methods pages 7-8
23	Transcripts returned	Were transcripts returned to participants for comment and/or correction?	Transcripts were emailed to the participants and they were given at least two weeks to review and adjust content to their transcript without limitations.<br><br>Results page 8

Domain 3: analysis and findings

Data analysis

Nr	Item	Guide question/description	
24	Number of data coders	How many data coders coded the data?	One researcher VB drafted the results and the reflection session was used to complement and validate principle findings.<br><br>Contributorship statement page 19
25	Description of the coding tree	Did authors provide a description of the coding tree?	No, a reflection session with experts was used to validate principle findings.<br><br>Research Methods page 8

For peer review only

26	Derivation of themes	Were themes identified in advance or derived from the data?	The transcript pieces were ordered by research question and themes were identified from these grouped transcript pieces.<br><br>Research Methods page 8-9
27	Software	What software, if applicable, was used to manage the data?	Excel was used to order and theme the transcript pieces.<br><br>Research Methods page 8
28	Participant checking	Did participants provide feedback on the findings?	Yes, the reflection session was used to complement and validate draft findings.<br><br>Research Methods page 8

Reporting

Nr	Item	Guide question/description	
29	Quotations presented	Were participant quotations presented to illustrate the themes / findings? Was each<br><br>quotation identified? e.g. participant number	One quote was used anonymously in the article and can be retraced by the researchers via the transcript identification number.<br><br>Results page 15
30	Data and findings consistent	Was there consistency between the data presented and the findings?	Yes, no contradicting statements were made in the interviews.<br><br>Results pages 9-16
31	Clarity of major themes	Were major themes clearly presented in the findings?	There was overall consensus in the reflection session on the final principal findings.<br><br>Results pages 9-16
32	Clarity of minor themes	Is there a description of diverse cases or discussion of minor themes?	In the reflection session examples were added to some statements to highlight nuances.<br><br>Results pages 9-16

For peer review only

Manuscript – Social care data and its fitness for integrated health and social care service governance: an exploratory qualitative analysis in the Dutch context

Véronique LLC Bos[1,2], Niek S Klazinga[1,2] and Dionne S Kringos[1,2]

[1] Department of Public and Occupational Health, Amsterdam UMC Location University of Amsterdam, Meibergdreef 9, Amsterdam, The Netherlands.

[2] Quality of Care, Amsterdam Public Health research institute, Amsterdam, The Netherlands.

Corresponding author

Véronique Bos

Department of Public and Occupational Health, Amsterdam UMC, University of Amsterdam

Van der Boechorststraat 7, 1081 BT Amsterdam, the Netherlands

Email: v.l.bos@amsterdamumc.nl

For peer review only

Supplemental Material 2: Dashboards desktop research & Data sources

Dashboards

Waar staat je gemeente (www.waarstaatjegemeente.nl)

Gemeentezorgspiegel (https://www.vektis.nl/gemeentezorgspiegel)

Regiobeeld (https://www.regiobeeld.nl/)

Labour Market Dashboards (https://www.werk.nl/arbeidsmarktinformatie/dashboards)

Early warning dashboard for possible debt (https://monitorvroegsignaleringshv.nl)

Data sources of included indicators

CBS - Arbeidsdeelname

CBS - Bevolkingsstatistiek

CBS - Bijstandsuitkeringenstatistiek

CBS - Enquête beroepsbevolking

CBS - Enquête sociale samenhang en Welzijn

CBS - Jeugdmonitor

CBS - Gezondheidsmonitor Volwassenen en Ouderen

CBS - Gemeentelijke Monitor Sociaal Domein

CBS - Monitor Abonnementstarief

CBS – Statistiek Re-integratie door Gemeenten

Divosa - Monitor Schuldhulpverlening

Divosa - Monitor Vroegsignalering Schulden

GGD-GHOR

RIVM

Vektis

For peer review only

Manuscript – Social care data and its fitness for integrated health and social care service governance: an exploratory qualitative analysis in the Dutch context

Véronique LLC Bos[1,2], Niek S Klazinga[1,2] and Dionne S Kringos[1,2]

[1] Department of Public and Occupational Health, Amsterdam UMC Location University of Amsterdam, Meibergdreef 9, Amsterdam, The Netherlands.

[2] Quality of Care, Amsterdam Public Health research institute, Amsterdam, The Netherlands.

Corresponding author

Véronique Bos

Department of Public and Occupational Health, Amsterdam UMC, University of Amsterdam

Van der Boechorststraat 7, 1081 BT Amsterdam, the Netherlands

Email: v.l.bos@amsterdamumc.nl

Supplemental Material 3 Translated interview guide

Thank you for your time

Going through informed consent form – when agreed, proceed

Affirm the scope of the research: Wet Maatschappelijke Ondersteuning, Participatiewet, Wet Gemeentelijke Schuldhulpverlening

- What data do you collect?
  - What is the purpose of your data collection?
  - Is there underlying legislation, standardization or other agreements or confinements as to how to manage the data?
- Data input points (potential legislation/standardization at entry point)
  - Who does the entry of the data?
  - With what purpose is the data entered in the system?
  - Is there underlying legislation, standardization or other agreements or confinements as to how to enter the data?
- Data sources and custodians
  - What database is the data collected in and who is owner of the database?
- Interoperability of sources
  - Does the database interact with other databases? If yes, with which other databases?

For peer review only

- Data management responsibilities, stakeholder inclusion, and regulators
    - How is data quality managed?
    - Are stakeholders included in quality management, transferring data to performance indicators, or other ways of data management?
    - What internal/external controls are executed for the database?
- Data outputs (information) and its users
    - What indicators are presented from the database?
    - Who uses the indicators to inform their decision making?
- In your perspective how can we make good use of social care data within the three laws mentioned for the benefit of integrated care? And what are potential hurdles to make use of this data?

For peer review only

Manuscript – Social care data and its fitness for integrated health and social care service governance: an exploratory qualitative analysis in the Dutch context

Véronique LLC Bos[1,2], Niek S Klazinga[1,2] and Dionne S Kringos[1,2]

[1] Department of Public and Occupational Health, Amsterdam UMC Location University of Amsterdam, Meibergdreef 9, Amsterdam, The Netherlands.

[2] Quality of Care, Amsterdam Public Health research institute, Amsterdam, The Netherlands.

Corresponding author

Véronique Bos

Department of Public and Occupational Health, Amsterdam UMC, University of Amsterdam

Van der Boechorststraat 7, 1081 BT Amsterdam, the Netherlands

Email: v.l.bos@amsterdamumc.nl

Supplemental Material 4 Interviewee list with background/expertise

organization	WHY?
VNG GMSD	Custodian of the Municipal Monitor of the Social Domain Dashboard and data.
VNG voorspelmodel	Custodian of the Social Care Act (WMO) prediction model
Divosa	Knowledge organisation supporting municipalities
Municipality of Amsterdam	Data team of the municipality
Stichting Inlichtingenbureau	Data processing service organisation established by the Ministry of Social Affairs and Employment (SZW) processing data about citizens who use social care schemes and facilities.
Vektis	Custodian of the Gemeentezorgspiegel (municipal care dashboard)
De Friesland	Health care insurer in the Friesland region working on integrated care projects using data.
RIVM (Regiobeeld)	Custodian of Regiobeeld

For peer review only

Sociaal Werk Versterkt	Representative of social workers
De Sociale Maatschap	Social care provider in de Amsterdam Noord region
Ketenbureau i-sociaal Domein	Data processing service organisation which facilitates and supports municipalities and healthcare providers in, among other things, simplifying the process from purchasing and tendering to accountability and reducing administrative burdens.
ZorgInstituut	Custodians of the i-standards (e.g. i_WMO standard)
BKR	Custodian credit registry (BKR)
LCR	National representatives for clients in social care

For peer review only

Manuscript – Social care data and its fitness for integrated health and social care service governance: an exploratory qualitative analysis in the Dutch context

Véronique LLC Bos[1,2], Niek S Klazinga[1,2] and Dionne S Kringos[1,2]

[1] Department of Public and Occupational Health, Amsterdam UMC Location University of Amsterdam, Meibergdreef 9, Amsterdam, The Netherlands.

[2] Quality of Care, Amsterdam Public Health research institute, Amsterdam, The Netherlands.

Corresponding author

Véronique Bos

Department of Public and Occupational Health, Amsterdam UMC, University of Amsterdam

Van der Boechorststraat 7, 1081 BT Amsterdam, the Netherlands

Email: v.l.bos@amsterdamumc.nl

Supplemental Material 1 COREQ 32-item checklist

Domain 1: Research team and reflexivity

Personal Characteristics

Nr	Item	Guide question/description	Answer and where to find in manuscript
1	Interviewer/facilitator	Which author/s conducted the interview or focus group?	Interviews: VB<br><br>Reflection session: NK, DK and VB<br><br>Contributorship statement page 19
2	Credentials	What were the researcher's credentials? E.g. PhD, MD	VB: BSc, MA<br><br>NK: Full Professor, MD<br><br>DK: Associate Professor<br><br>Contributorship statement page 19
3	Occupation	What was their occupation at the time of the study?	VB: PhD candidate<br><br>NK: Professor of Social Medicine

			DK: Associate Professor and Principal Investigator & Educator<br><br>Contributorship statement page 19
4	Gender	Was the researcher male or female?	VB: female<br><br>NK: male<br><br>DK: female<br><br>Contributorship statement page 19
5	Experience and training	What experience or training did the researcher have?	Experienced in interviews, reflection sessions and qualitative research<br><br>Contributorship statement page 19

Relationship with participants

Nr	Item	Guide question/description	
6	Relationship established	Was a relationship established prior to study commencement?	Before the start of the study, the study and the research team were introduced to the interviewees and reflection session experts via email.<br><br>Research Methods pages 7-8
7	Participant knowledge of the interviewer	What did the participants know about the researcher? e.g. personal goals, reasons for doing the research	In the communication to participants the background of the interviewer was listed, as well as research goals.<br><br>Research Methods pages 7-8
8	Interviewer characteristics	What characteristics were reported about the interviewer/facilitator? e.g. Bias, assumptions, reasons and interests in the research topic	The credentials, occupation and department of the research team was communicated.<br><br>Research Methods pages 7-8

For peer review only

Domain 2: study design

Theoretical framework

Nr	Item	Guide question/description	
9	Methodological orientation and Theory	What methodological orientation was stated to underpin the study? e.g. grounded theory, discourse analysis, ethnography, phenomenology, content analysis	A constructivism explorative approach with inductive reasoning was used. Research Methods page 6

Participant selection

Nr	Item	Guide question/description	
10	Sampling	How were participants selected? e.g. purposive, convenience, consecutive, snowball	Purposive sampling based on the desktop research and participant referral Research Methods page 7
11	Method of approach	How were participants approached? e.g. face-to-face, telephone, mail, email	The participants were approached via email, phone and LinkedIn. Research Methods page 7
12	Sample size	How many participants were in the study?	18 participants in the interviews, and 10 participants in the reflection session. Results page 9
13	Non-participation	How many people refused to participate or dropped out? Reasons?	3 participants dropped out without stating a reason, 1 participant refused to participate due to prioritisation issues at that moment Research Methods page 7

Setting

Nr	Item	Guide question/description	
14	Setting of data collection	Where was the data collected? e.g. home, clinic, workplace	Most interviews were conducted digitally or in the work environment of the interviewee.

For peer review only

			The reflection session was held digitally.<br><br>Research Methods page 7
15	Presence of non-participants	Was anyone else present besides the participants and researchers?	No<br><br>Contributorship statement page 19
16	Description of sample	What are the important characteristics of the sample? e.g. demographic data, date	Participants from different levels of the health care system (micro, meso, macro) and different perspectives (clients, providers, data processors, policy advisors) were included in the sample.<br><br>Research Methods page 7

Data collection

Nr	Item	Guide question/description	
17	Interview guide	Were questions, prompts, guides provided by the authors? Was it pilot tested?	The interview guideline was grounded in the Health care performance intelligence pyramid and is provided in appendix 3. It was not pilot tested.<br><br>Research Methods page 7
18	Repeat interviews	Were repeat interviews carried out? If yes, how many?	No, however some questions that could not be answered during interviews were answered at a later moment in time or corrected in the transcript by the interviewee.<br><br>Research Methods page 8
19	Audio/visual recording	Did the research use audio or visual recording to collect the data?	Interviews and the reflection session were audio recorded<br><br>Research Methods page 8

For peer review only

20	Field notes	Were field notes made during and/or after the interview or focus group?	Notes were taken by two researchers (VB and DK) during the reflection session.<br><br>Contributor statement page 19
21	Duration	What was the duration of the interviews or focus group?	The duration of the interviews were between 33-60 minutes.<br><br>Research Methods page 7
22	Data saturation	Was data saturation discussed?	Participants could adjust content to their transcript without limitations and the reflection had the function to validate and if needed complement draft findings.<br><br>Research Methods pages 7-8
23	Transcripts returned	Were transcripts returned to participants for comment and/or correction?	Transcripts were emailed to the participants and they were given at least two weeks to review and adjust content to their transcript without limitations.<br><br>Results page 8

Domain 3: analysis and findings

Data analysis

Nr	Item	Guide question/description	
24	Number of data coders	How many data coders coded the data?	One researcher VB drafted the results and the reflection session was used to complement and validate principle findings.<br><br>Contributorship statement page 19
25	Description of the coding tree	Did authors provide a description of the coding tree?	No, a reflection session with experts was used to validate principle findings.<br><br>Research Methods page 8

For peer review only

26	Derivation of themes	Were themes identified in advance or derived from the data?	The transcript pieces were ordered by research question and themes were identified from these grouped transcript pieces.<br><br>Research Methods page 8-9
27	Software	What software, if applicable, was used to manage the data?	Excel was used to order and theme the transcript pieces.<br><br>Research Methods page 8
28	Participant checking	Did participants provide feedback on the findings?	Yes, the reflection session was used to complement and validate draft findings.<br><br>Research Methods page 8

Reporting

Nr	Item	Guide question/description	
29	Quotations presented	Were participant quotations presented to illustrate the themes / findings? Was each<br><br>quotation identified? e.g. participant number	One quote was used anonymously in the article and can be retraced by the researchers via the transcript identification number.<br><br>Results page 15
30	Data and findings consistent	Was there consistency between the data presented and the findings?	Yes, no contradicting statements were made in the interviews.<br><br>Results pages 9-16
31	Clarity of major themes	Were major themes clearly presented in the findings?	There was overall consensus in the reflection session on the final principal findings.<br><br>Results pages 9-16
32	Clarity of minor themes	Is there a description of diverse cases or discussion of minor themes?	In the reflection session examples were added to some statements to highlight nuances.<br><br>Results pages 9-16

For peer review only

Social care data and its fitness for integrated health and social care service governance: an exploratory qualitative analysis in the Dutch context

Journal:	*BMJ Open*
Manuscript ID	bmjopen-2023-078390.R2
Article Type:	Original research
Date Submitted by the Author:	22-Mar-2024

Complete List of Authors:	Bos, Véronique; Amsterdam UMC Location AMC, Department of Public and Occupational Health; Amsterdam Public Health Research Institute, Quality of Care<br>Klazinga, Niek; Amsterdam UMC Locatie AMC, Department of Public and Occupational Health; Amsterdam Public Health Research Institute, Quality of Care<br>Kringos, D; Amsterdam UMC Location AMC, Department of Public and Occupational Health; Amsterdam Public Health Research Institute, Quality of Care

**Primary Subject Heading**:	Health policy
Secondary Subject Heading:	Health informatics, Public health, Qualitative research, Evidence based practice
Keywords:	Information management < BIOTECHNOLOGY & BIOINFORMATICS, Health policy < HEALTH SERVICES ADMINISTRATION & MANAGEMENT, Organisation of health services < HEALTH SERVICES ADMINISTRATION & MANAGEMENT, Information Extraction < Information Storage and Retrieval, QUALITATIVE RESEARCH, SOCIAL MEDICINE

SCHOLARONE™
Manuscripts

I, the Submitting Author has the right to grant and does grant on behalf of all authors of the Work (as defined in the below author licence), an exclusive licence and/or a non-exclusive licence for contributions from authors who are: i) UK Crown employees; ii) where BMJ has agreed a CC-BY licence shall apply, and/or iii) in accordance with the terms applicable for US Federal Government officers or employees acting as part of their official duties; on a worldwide, perpetual, irrevocable, royalty-free basis to BMJ Publishing Group Ltd ("BMJ") its licensees and where the relevant Journal is co-owned by BMJ to the co-owners of the Journal, to publish the Work in this journal and any other BMJ products and to exploit all rights, as set out in our licence.

The Submitting Author accepts and understands that any supply made under these terms is made by BMJ to the Submitting Author unless you are acting as an employee on behalf of your employer or a postgraduate student of an affiliated institution which is paying any applicable article publishing charge ("APC") for Open Access articles. Where the Submitting Author wishes to make the Work available on an Open Access basis (and intends to pay the relevant APC), the terms of reuse of such Open Access shall be governed by a Creative Commons licence – details of these licences and which Creative Commons licence will apply to this Work are set out in our licence referred to above.

Other than as permitted in any relevant BMJ Author's Self Archiving Policies, I confirm this Work has not been accepted for publication elsewhere, is not being considered for publication elsewhere and does not duplicate material already published. I confirm all authors consent to publication of this Work and authorise the granting of this licence.

For peer review only

Social care data and its fitness for integrated health and social care service governance: an exploratory qualitative analysis in the Dutch context

Véronique LLC Bos[1,2], Niek S Klazinga[1,2] and Dionne S Kringos[1,2]

[1] Department of Public and Occupational Health, Amsterdam UMC Location University of Amsterdam, Meibergdreef 9, Amsterdam, The Netherlands.

[2] Quality of Care, Amsterdam Public Health research institute, Amsterdam, The Netherlands.

Corresponding author

Véronique Bos

Department of Public and Occupational Health, Amsterdam UMC, University of Amsterdam

Van der Boechorststraat 7, 1081 BT Amsterdam, the Netherlands

Email: v.l.bos@amsterdamumc.nl

Supplemental Material

Supplemental Material 1: Research checklist: COREQ 32-item checklist

Supplemental Material 2: Sources desk top research

Supplemental Material 3: Translated interview guide

Supplemental Material 4: Interviewee list with background/expertise

For peer review only

Abstract (298 words)

INTRODUCTION: To date, little is known on how social care data could be used to inform performance-based governance to accelerate progress towards integrated health and social care.

OBJECTIVES AND DESIGN: This study aims to perform a descriptive evaluation of available social care data in the Netherlands and its fitness for integrated health and social care service governance. An exploratory mixed method qualitative study was undertaken based on desktop research (forty-one included indicators), semi-structured expert interviews (thirteen interviews including eighteen experts) and a reflection session (ten experts).

SETTING: The Netherlands; social care is care provided in accordance with the Social Support Act, the Participation Law, and the Law for Municipal debt-counselling.

RESULTS: This study found six current uses for social care data: 1) communication and accountability, 2) monitoring social care policy, 3) early warning systems, 4) controls and fraud detection, 5) outreaching efforts, and 6) prioritisation. Further optimization should be sought through: standardization, management of data exchange across domains, awareness of the link between registration and financing, strengthening the overall trust in data sharing. The study found five ways the enhanced social care data could be used to improve the governance of integrated health and social care services: 1) cross-domain learning and cooperation (e.g. through benchmarks), 2) preventative measures and early warning systems, 3) give insight regarding the quality and effectivity of social care in a broader perspective, 4) clearer accountability of social care towards contracting parties and policy, 5) enable cross-sector data-driven governance model.

CONCLUSION: Although there are several innovative initiatives for the optimization of the use of social care data in the Netherlands, the current social care data landscape and management is not yet fit to support the new policy initiatives to strengthen integrated health and social care service governance. Directions for addressing the shortcomings are provided.

Strengths and limitations

- In our desk-top research we used an explorative approach and publically available data sources. This can have biased our findings towards finding more data sources aligned with government roles.
- We limited our scope to data sources with a national representation which can have biased results towards finding more aggregate data.
- We minimized these biases by including experts from different levels (micro, meso and macro) and experts from a diverse social care data processing and use background in the interviews and reflection session to enrich the desktop study and validate results.

For peer review only

- Public sources in social care data like the dashboards and monitors used in this study are constantly developing, thus the results of the study are subject to change over time.

Keywords (3-6)

social care data, performance information, Integrated health services, the Netherlands, informed decision making, health care governance

For peer review only

Introduction (background & problem statement)

Sound and reliable health and social care information at the right place and at the right time is essential for effective decision making in health systems [1-3]. Integrated health services, described by the WHO as "health services that are managed and delivered in a way that ensures people receive a continuum of health promotion, disease prevention, diagnosis, treatment, disease management, rehabilitation and palliative care services, at the different levels and sites of care within the health system, and according to their needs throughout their life course"(WHO, 2015), has become an important focus of many health system reforms. As a consequence, performance information presented in the traditional siloes of public health data, health care data and social care data form a hindrance for effective governance across the domains of health promotion, disease prevention, diagnosis, treatment, disease management, rehabilitation and palliative care services [4, 5]. Challenges in financing (rising (complexity in) health and social care needs and costs), personnel (lack of care professionals) and society (e.g. pressure on solidarity) are jeopardizing the sustainability of the health care system [6].

The COVID-19 pandemic has highlighted that in order to effectively monitor and govern our health systems, data from a broad range of sources using demographics, social care data, health care data, and public health data needs to be systematically integrated. This was demonstrated by the need to monitor information in coherence and including social, behavioural and economic perspectives next to epidemiologic considerations.[7]. Countries worldwide are struggling with integrating these diverse types of data to establish comprehensive performance intelligence for decision making. Despite data rich health systems, these systems are information poor, indicating an unfulfilled potential of data and digital technologies [8]. Additionally, the COVID-19 pandemic has increased the pressure on the often already overstretched health care services and national budgets globally [9]. In order to reduce this pressure policy makers are shifting their attention to health promotion and disease prevention. Strengthening social care is part of this agenda. Investments in social care (e.g. housing, income support, nutrition support, care coordination, and community outreach) can have a positive impact on the health of citizens and health care service needs and use [10].

Countries and regions worldwide are investing in an integrated health and social care data infrastructure. The main purposes of the data infrastructures differ from primary uses of data (e.g. supporting clinical practice) to facilitate a vast range of secondary data uses (e.g. research, policy, governance) on regional or national levels. For instance, Finland's Sotkanet, a national health and social care data infrastructure, has more comprehensive data available to inform their decision making from

For peer review only

a broader welfare perspective [11]. The affiliated Oulu Self Care Service shows that an integrated data system can also function as an enabler and improve chronic care health outcomes and efficiency through shared use of health and social care data among care providers [12]. New Zealand's integrated health infrastructure (IDI) illustrates that cross-cutting data can give insights needed to govern effectively throughout the health and welfare system [13, 14]. This national integrated health infrastructure was for example used for descriptive research to characterize adolescents who experience poor health outcomes [15]. It was also used to define cohorts in the existing population; one example was a cohort of people with chronic diseases to define effects on work and income [16]. Thus, linking social care data to health care data shows potential for integrated governance, but there are also known pitfalls. A Canadian study linking administrative social assistance data to health care data warns for potential biases due to linkage errors [17]. A study in Dundee, Scotland found that linkage between health and social care data faced challenges around data linkage (e.g. use of a shared identifier across organisations), data analysis (e.g. understanding missing data), and the need for tacit knowledge (e.g. people understanding the data, its limitations and what it represents) limiting the full exploitation [18]. The Integrated Children's System in the UK initially followed a top-down managerial approach emphasizing on accountability of institutional risk instead of user-centeredness which had a detrimental effect on professional autonomy [19]. These examples show that a thorough understanding of social care data, its characteristics and use are needed to exploit its potential to gain actionable insights.

The motto of the Dutch Ministry of Health, Welfare and Sport is "Everyone healthy, fit and resilient". In the Netherlands multi-stakeholder policy agreements on strategic directions are considered a classical tool for governing the health- and welfare sectors. The latest policy changes aim to transform an illness insurance system into a health insurance system. In 2022, Dutch stakeholders in the health and welfare sectors launched multiple integrated policy agreements and programmes to facilitate a more integrate delivery of health and social care services: the Integrated Care Agreement (IZA), the Healthy and Active living Agreement (GALA), the Housing, Support and Care for the Elderly Programme (WOZO) and the Future-proof Care and Welfare Labour Market Programme [20-23]. In these initiatives, cooperation and integration of health and social care services and prevention efforts are highlighted as important ways forward to subdue the pressure on the healthcare system and its staff. The Netherlands has many (N: > 450) care partnerships [24]. These are often regional initiatives and alliances that are strengthening their provision of integrated health and care services for better outcomes [25]. The bandwidth of data-driven action in these collaborations is broad. Some collaborations see it as a need to have, others as a nice to have [24]. In 2015, the decentralisation of social care responsibilities increased the municipal responsibilities towards vulnerable individuals and

For peer review only

populations. Efforts in integrated care are particularly aimed at for vulnerable individuals and populations[20-22, 26, 27]. With a high political and regional interest in integrating health and social services, the Netherlands is a relevant country for an explorative study on social care data and its fitness for integrated health and social care service governance.

General aims and objectives

This study aims to perform a descriptive evaluation of the landscape of social care data in the Netherlands, its current use and how it can be improved. It attempts to explore the fitness for purpose and use of social care data to contribute to integrated health and social care service governance.

Research questions

1. What are the current uses of social care data in the Netherlands, and how can the usability of this data be enhanced?
2. In what ways can enhanced social care data contribute to the governance of integrated health and social care services?

Research Methods

Research design and scope

A constructivism exploratory qualitative study design [28] was employed using: 1) desktop research, 2) semi-structured expert interviews and 3) a reflection session. The study conforms to the Consolidated Criteria for Reporting Qualitative Studies, the 32-item checklist for this study can be found in Supplemental Material 1. Our research design was grounded in the Health care performance intelligence pyramid [1]. The hierarchical pyramid defines how measurement through data collection and its translation to indicators is at the base of governance and management through information and knowledge. Action and utilisation is the ultimate end goal and needs its own translation from information and knowledge to practice. Each of these steps needs to consider the regulatory, organisational, political and cultural context.

The scope of this research is social care as provided within the municipal responsibilities in the Netherlands through: 1) the Social Support Act (Wet Maatschappelijke Ondersteuning, WMO); regulating municipal responsibility for supplementing citizens in their care need, 2) the Participation Law (Participatiewet); regulating municipal responsibility for supporting citizens in participating in the society, and 3) the Law for Municipal debt-counselling (Wet Gemeentelijke Schuldhulpverlening);

For peer review only

regulating municipal responsibility for supporting citizens in financial need. As the focus of this study is on the adult population we have excluded social care as provided by the Youth Act (Jeugdwet); regulating the preventative and mental health care for children. As social care was decentralised to municipal level in 2015 in the Netherlands, we have only included documents from beyond this time point in our desktop research.

Patient and Public Involvement

Clients, patients and the public have not been involved in the design or conduct of the study. Social care clients were represented in the interviews and the reflection session by the National (social care) Client Council. The results of the study will be disseminated through our network and shared with all study participants.

Data collection and coding

Our data collection took place from January 2022 to October 2022. The desktop research aimed to get an overview of publicly available social care indicators. We used two starting points: 1) the website vzinfo.nl of the National Institute for Public Health and the Environment commissioned by the Ministry of Health, Welfare and Sport, which provides an overview and metadata of available public health and care information and 2) a social care stakeholder analysis done by Driesten and Blink in 2021 [29]. Data included in this study was either collected on a national level, or purposefully sampled to represent the national population. Data(sets) that were collected only for a single organisation/municipality or region were excluded from this study. We collected all described indicators from dashboards and reports found via these two starting points that met the scope of this research. An overview of all the collected dashboards and reports included in the desktop research and their references can be found in Supplemental Material 2.

The expert interviews aimed to complement the desktop research and highlight current uses of social care data and indicators within the social care domain, to describe ways to optimize social care data (infrastructure), and to highlight potential uses for integrated health and social care service governance. We purposively sampled experts based on the sources found in the desktop research, and gave them the opportunity to refer to other experts in the field of social care data in the Netherlands. The participants were approached via email, phone and LinkedIn. We aimed to have at least two representatives from each level of the health care system: micro (social care clients and care professionals), meso (data custodians and data processing organizations), and macro (policy and legal experts) levels. Most interviews were organized digitally in the aftermath of the COVID-19 pandemic and the duration of the interviews was between thirty-three and sixty minutes. Three approached

For peer review only

participants dropped out without stating a reason, one approached participant could not to participate due to prioritisation issues at that moment. The interview guideline (Supplemental Material 3) was grounded in the Health care performance intelligence pyramid. Before the start of the study, research goals and the research team (department, credentials and occupation) were introduced to the interviewees via email. With the written informed consent of participants, all interviews were audio-recorded and transcribed. Transcripts were emailed to the participants and they were given at least two weeks to review and adjust the content of their transcript without limitations. The validated transcripts were used for the thematic analysis.

The reflection session was organized on 12 October 2022 with the following aims: 1) to validate the draft results from the desktop research and semi-structured interviews and 2) to consolidate key findings. It was organized digitally in the aftermath of the COVID-19 pandemic. First, the study team presented the draft results. Second, participants shared their general reflections on the draft results. Finally, the following questions were discussed in-depth: 1) Is the outlined landscape of available social care data and indicators, its management and its use recognisable?, 2) How can we optimize the use of available social care data within the social care domain?, 3) How can social care data contribute to public health and health care? And vice versa?, and 4) How can social care data be used in governing towards regional integrated care provision? The reflection session was audio recorded with the verbal informed consent of all participants. The draft results from the desktop study and expert interviews were shared with the participants in advance.

<u>Data analysis</u>

To answer research question one, we used information retrieved from desktop research and interviews. First, we constructed an overview of all dashboards and documents collected with their data custodian and a qualitative description of their content (Supplemental Material 2). Then we abstracted all indicators within the scope of this research and grouped them in their legal silos. Finally, we categorized them into the following subgenres: input, process and output, outcome, and impact and allocated source types (administrative, medical record, registry, survey, direct observation) to each indicator. Transcript pieces that described available social care data in the Netherlands were used to enrich the desktop research by adding additional data sources. We selected and then grouped all pieces of the transcripts from the interviews that described social care data in the Netherlands, its current use and how it can be improved. Then we analysed the transcript pieces using an analytic induction process using key words and statements to define how social care data is currently used and can be improved.

For peer review only

To answer research question two, pieces of the interview transcripts were grouped that relate to the current and potential use of social care data to improve integrated health and social care service governance. Then an analytic induction process using key words and statements was used to define how social care data was used for integrated health and social care service governance. The draft results of research question one and two were presented, enriched, clarified and verified in the reflection session.

Results

Characteristics of the data sources and informants

The sources included in the desktop research can be found in Supplemental Material 2. Thirteen interviews were conducted with a total of eighteen experts including representatives for social care clients and providers , representatives from the municipalities, social care administrative data processors, owners of social care dashboards and indicator reports and experts from the health care insurance data field with experience in integrated care projects. Three participants made minor changes to their interview transcript. The changes contained clarifications of the content and additions to the content. No changes to the transcripts were made that affected the results. The interviewee list with background/expertise can be found in Supplemental Material 4. The reflection session included ten experts on social care data, including representatives for social care clients (n=1) and providers (n=2), representatives from the municipalities (n=2), social care administrative data processors (n=4, from 3 different organizations and working with different social care data), and an expert from the health care insurance data field with experience in integrated care projects (n=1).

What social care data is available in the Netherlands and how is it used?

Measuring social care data: data collection and Indicators

The most common data source types used to populate the included social care indicators were administrative data (including the needs assessment done for administrative purposes) and surveys (often samples representative to national population). Indicators on use and costs of services were most common and most often reported on municipal or neighbourhood level. These indicators were often updated once or twice a year. There is a diversity in measurement instruments to measure an individual's needs for social care. However, there is a national guideline for a needs assessment obligatory in order to receive the social care provided. Prediction models (e.g. the Social Support Act prediction model) are available. However, one expert mentioned that policymakers find such models difficult to apply due to the lack of data driven personnel and a data driven work culture. Policy

For peer review only

makers are also hesitant to use these models as they are by definition incorrect, as they only model reality and can prove to be incorrect in hindsight.

The use of client reported data for decision making is very limited in the social care domain. There is a legal obligation in the Social Support Act for municipalities to carry out a national client experiences measure. Until 2021 this measure consisted of 10 nationally defined questions divided in three themes: 1) access to services, 2) quality of the services provided, and 3) the effect on the independence for the client and his/her participation in society. However, since 2021, due to suboptimal use of the measure, municipalities are free to adapt the client experience measure to their needs, either using the 10 defined questions, adding additional questions or using their own survey format. Experts state that this tailoring of the survey by municipalities might be detrimental to cross-municipality comparisons. Besides the national client experience measure, client stories are occasionally used to represent clients' voices to complement the data provided, this is relatively customary in the municipal reporting on social care.

The tables below give an overview of all included indicators and their defined indicator and data source type per legal silo.

For the Social Support Act we included 17 indicators (table 1). Most were sourced from survey data or administrative data and gave insight on (potential) inputs or processes and outputs.

Table 1 Social care indicators included in the desktop study within scope of the Social Support Act

Indicator	Type of indicator	Type of source
Trend in the number of potential Informal carers	(potential) Input	survey data
% Vulnerable population	(potential) Input	administrative / survey / medical record data
% of people with one or more limitation in daily functioning	(potential) Input	survey data
% of people with one or more restrictions in their mobility	(potential) Input	survey data
% of people with restrictions in their eye sight	(potential) Input	survey data
% of people with hearing disabilities	(potential) Input	survey data
Budgeted and actual costs for Social Support Act	Input	administrative
Municipal policy (only customized provisions, mainly customized provisions, as many general as customized provisions, mainly general provisions, only general provisions) of Social Support Act care provision	Input	survey data
Ratio between households with and without care provision	Process & Output	administrative
Relative number of households with provisions of care and support by number of provisions	Process & Output	administrative
Relative number of households with provisions of care and support by number of provisions per domain	Process & Output	administrative

For peer review only

Trend number of inhabitants with Social Support Act care provision	Process & Output	administrative
Relative number of inhabitants with Social Support Act care provision by category	Process & Output	administrative
Number of inhabitants with Social Support Act care provision	Process & Output	administrative
Relative number of inhabitants in sheltered housing by region	Process & Output	survey data / administrative
Number of terminated Social Support Act provisions by reason (total, deceased, planned/anticipated termination, move to other municipality, other/unknown)	Process & Output	administrative
Qualitative reporting of the WMO Client experience outcomes	Outcome	survey data

For the Participation law we included 19 indicators (table 2). All were sourced from survey data or administrative data and most gave insight on processes and outputs or outcomes.

Table 2 Social care indicators included in the desktop study within scope of the Participation law

Indicator	Type of indicator	Type of source
Trend in pension age	Input	survey data
Ratio between households with and without care provision	Process & Output	administrative
Relative number of households with provisions of care and support by number of provisions	Process & Output	administrative
Relative number of households with provisions of care and support by number of provisions per domain	Process & Output	administrative
Number of inhabitants with a welfare benefit	Process & Output	administrative
Number of inhabitants with a welfare benefit by municipality and reference group	Process & Output	administrative
Number of inhabitants with a reintegration facility	Process & Output	administrative
Relative number of inhabitants with a reintegration facility by municipality and reference group	Process & Output	administrative
Number of unemployed inhabitants in working age	Outcome	survey data
% inhabitants participating in formal work, volunteer work and informal care by sex	Outcome	survey data
% of persons with/without health problems with formal work by age categories	Outcome	survey data
% of the Formal work population with/without health problems by age categories	Outcome	survey data
% of net formal work participation by age category and sex	Outcome	survey data
Number of inhabitants of the formal workers working part-time by sex	Outcome	survey data
% Unemployment by age category and sex	Outcome	survey data
Trend % net formal work participation by two age categories and sex	Outcome	survey data
Trend % unemployment by age category and sex	Outcome	survey data
% Unemployed young professionals	Outcome	survey data

For peer review only

SES-WOA score (financial prosperity, level of education and recent employment history of private households)	Impact	administrative

For the Law for Municipal debt-counselling we included 9 indicators (table 3). All were sourced from survey data or administrative data and most gave insight on processes and outputs.

Table 3 Social care indicators included in the desktop study within scope of the Law for Municipal debt-counselling

Indicator	Type of indicator	Type of source
Municipal capacity to provide debt counselling services (enough capacity for demand, enough capacity for demand, but sustainability for the future is under pressure, not enough capacity for demand)	Input	survey data
Factors that determine the manner in which early warning signals for over indebtedness are followed up (type of notification, debt size, type of non-payment, age, composition of the household, time passed since notification, postal code)	Input	administrative / survey data
Number of applications for municipal debt counselling services	Process & Output	survey data
Qualitative reporting by cooperating municipalities on the (change in) target group for municipal debt counselling services	Process & Output	survey data
Number of early warning signals (one non-payment from one specific provider on one home address) per 1000 inhabitants per municipality	Process & Output	administrative
Number of early warning notifications (multiple signals at one address) per municipality	Process & Output	administrative
Number of contacts (defined as contact with a reaction, e.g. reply to email, opening the door, answering the phone call) resulting from early warning signals	Process & Output	administrative
% of inhabitants accepting the offer for debt counselling services	Process & Output	Administrative
SES-WOA score (financial prosperity, level of education and recent employment history of private households)	Impact	administrative

Governance and management in social care data: rules for translation to information and knowledge

National administrative registration standards are in place, for example the i-WMO standard. However, municipalities have the legal mandate for the social care provision. This mandate also includes how to structure and use (administrative) social care data. This makes national level benchmarking between municipalities difficult, due to a high variability in interpretation and use of

For peer review only

the available national standards. The diversity of payment models within and across municipalities also impact the way municipalities structure this data, making this data only interpretable if: 1) within a known context (for example on municipal level) or 2) aggregated to very general terms where this variation is diminished. Our sources also showed a big variety between municipalities in data processing power, ranging from own data processing departments to support policy decisions to the minimal data processing needed for accountability purposes. This variety in data processing power also exists between social care provider organizations.

Legislation on the use of social care data mainly covers accountability of social provisions (fraud and supervision), the use of the citizen service number for identification, and permission required for use of personal data (privacy). Multiple public and private parties can fulfil the role of data processor in social care in the Netherlands, however often under municipal authority. But the use of social care data for quality improvement of care provision by integrated provider networks, research or policy action is not defined in the legislation. Thus, a weighing between respecting privacy of personal data on the one hand and having data available to deliver adequate social care services or take timely policy action on the other hand, cannot be made.

Utilisation of social care data: what is done with the acquired knowledge through data

Social care data is mostly used in the *communication and accountability* between care providers and financers (e.g. official needs assessments or registration of start and end a care trajectory), and in *monitoring social care policy* on municipal and national levels (e.g. Monitor on adult health and health of the elderly). There is an *early warning system* on over-indebtedness using non-payments of housing, utilities and health care insurance to alert municipalities to a potential demand in social care services to prevent over-indebtedness. According to one of the care organisations interviewed this proactive exchange of information has led to the prompt identification of individuals requiring services, thereby minimizing the need for involuntary out-of-house placements in the serviced district. Nevertheless, additional insights from interviewed experts underscored that in numerous regions, the transition from data availability to utilizing that data for identification and subsequent action by municipalities, necessary for achieving impact, still requires improvement. Data alone does not lead to action.

Traditionally, data in the social care domain has been used for *controls and fraud detection*, which has led to a distrust in data exchange in the social care domain from the care provider and client perspective. Examples of information used for controls and fraud detection mentioned by interviewees were: 1) financial information on care providers to guide the municipal purchasing risk management strategy and 2) information on which clients have access to Long Term Care Act

For peer review only

resources to check whether the Social Support Act resources given are still relevant. Some recent examples rethink this approach, like the *outreaching efforts* of the Social Insurance Bank (SVB) to track and locate eligible citizens entitled to receive a pension, yet for reasons unknown, have not (yet) applied for it. We've identified an illustrative *prioritisation* case where a social work organization employed national demographic data in tandem with their client base to evaluate whether they are effectively directing their efforts toward the neighbourhood exhibiting the highest (predicted) need for debt counselling.

Improving the social care data use

Data: Standardization of registration

A high variation between municipalities in social care data, data infrastructure and processing (power) is the result of policy decentralization efforts aiming to provide care 'closer to home'. Efforts to standardize product codes have already yielded results (from 100.000 product codes in 2015 to about 3.000-4.000 product codes per law during the time of this research). However, some experts interviewed believe that more standardization in social care data, its infrastructure and its processing can increase comparability and decrease administrative burden on the long run for care provider organizations and municipalities alike. We found heterogeneous roles and responsibilities in the coordination and alignment of data processing in social care. It has become difficult to compare even similar indicators due to source and processing variability. Establishing a well-defined governance structure for social care data, along with a robust infrastructure and processing framework, could enhance the stability and interpretability of the available data for more effective utilization. Standardization initiatives can provide a coherent framework for data registration, thereby mitigating interpretation challenges associated with the registration process. Representatives from social care organizations emphasize the importance of considering that standardizing data registration does not imply a necessity to diminish regional variability in care delivery, especially when diverse regions exhibit distinct care needs.

Governance and management of social care data across domains and within clear roles for a societal purpose

Data processing is often operationalized per legal and financial silo. Experts state that in order to have a clear picture of the societal needs and impact of social care, insight in performance information across legal and financial silos is needed (e.g. the association between over-indebtedness and health care use). However, data use across domains is legally and politically restricted in the Netherlands. Additionally, cross-sector data sharing, including across private and public interests, may have more potential for *preventative measures*, but is politically sensitive. For

For peer review only

example, in the context of nationally organized debt counselling initiatives, it is noteworthy that study loans exhibit a higher average credit per person compared to consumer credits. However, it's essential to acknowledge that study loans, in contrast to consumer credits, face less transparent monitoring by credit providers, primarily due to political sensitivity. The implementation of these supplementary prevention options could yield positive effects, provided they operate within the requisite boundaries of a robust ethical and legal framework. This ensures alignment between data use and its intended societal purpose while avoiding any potential discrepancies.

Multiple experts state that legislation concerning data exchange and processing ought to prioritize explicit use and purpose rather than merely restricting (while simultaneously facilitating extensive) data processing and integration within specific financial or legal silos. The measurement of service effectiveness and affordability remains constrained, as does the availability of metrics aligned with newly articulated national policy goals, such as the Integrated Care Agreement. Currently, there is limited data on the well-being of citizens, including how to sustain healthy lives and identify potential risks to health.

Awareness of the association between registration and financing mechanisms

We found that indicators in social care frequently rely on administrative data sources, leading to a notable influence from financing structures and incentives on these indicators. One of the examples given in the interviews was in a Participation law indicator. When an individual transitions to a different role within the same company, the data records this as a job change or switch, with an "end of trajectory" and a subsequent "new beginning of trajectory" being registered. Leveraging this data to gain insights into stability in societal participation was inherently biased, revealing an overemphasis on short-term employer relations.

According to the experts in our study, financing on a population level remains more theoretical than practical. Nonetheless, a shift in financing models could potentially impact data availability within the social care domain. For example, there are municipalities that have started using clustered/population/performance contracts in contracting social care organisations. This means that some municipalities no longer are able to distinguish (parts of) the care provided into potentially relevant subgroups, thus registering entries under “other”, “unknown” or estimating percentages.

Improve the trust relationship between municipalities, care providers and clients

The complexity of the social care data infrastructure and processing compromises the transparency towards clients. This has consequences for the trust clients have in the processing of their data. As one expert puts it: “Unknown makes unloved.” This, together with the history of social care data uses

For peer review only

towards control and fraud purposes has deteriorated the relationship between social care financers (e.g. municipalities) and clients. To improve this relation a dialogue is needed between clients and social care financers to discuss the different perspectives and bring them together. Both parties must acquire the necessary skills to engage in this dialogue effectively. Additionally, illustrating the processes involved in data exchange and showcasing the potential benefits, as well as the actual outcomes and impact it brings to society, can enhance this relationship. An alternative approach involves shifting the utilization of data from control and fraud detection purposes to prioritizing citizen rights. Data could then be employed to identify individuals entitled to specific social care provisions but are not currently receiving them.

Potential uses for social care data to improve the governance in integrated care practice and policies

Experts state that the enhanced social care data can contribute to the governance of integrated health and social care services . Five potential uses were stated: 1) Cross-domain data (including social care data) can facilitate cross-domain learning and cooperation (e.g. through benchmarks), 2) Integrating social care data with population demographics, welfare, public health, and health care data can facilitate preventative measures and create early warning systems, 3) It can give insight regarding the quality and effectivity of social care in a broader perspective, 4) Better social care insights can enable a clearer accountability of social care towards contracting parties and policy, and 5) It can facilitate parties in a more data-driven governance model.

Discussion

Principle findings

This study aimed to investigate the current applications of social care data in the Netherlands, identify areas for improvement, and assess how enhanced social care data can contribute to the governance of integrated health and social care services. We found that there is a rich array of social care data sources and information applications in the Netherlands such as public dashboards and policy monitors. Six current uses for social care data were identified: 1) communication and accountability, 2) monitoring social care policy, 3) early warning systems, 4) controls and fraud detection, 5) outreaching efforts, and 6) prioritisation. However, the present state of data and information in social care restricts its use for integrated care governance. Enhancing the data involves initiatives in standardization, effective management of data exchange across domains, recognizing the connection between registration and financing, and bolstering societal trust in data sharing overall. Five potential ways were identified to use this enhanced data to improve the

For peer review only

governance in integrated health and social care services: 1) cross-domain learning and cooperation (e.g. through benchmarks), 2) preventative measures and early warning systems, 3) give insight regarding the quality and effectivity of social care in a broader perspective, 4) clearer accountability of social care towards contracting parties and policy, 5) enable cross-sector data-driven governance model.

Strengths and weaknesses of the study

This study is conducted within the Dutch context but can serve as inspiration for other high-income countries transitioning from a fragmented healthcare regulated market to regional governance of integrated care. In our desk-top research we used an explorative approach and publicly available data sources. This can have biased our findings towards finding more data sources aligned with government roles. We limited our scope to data sources with a national representation which can have biased results towards finding more aggregate data. We minimized these biases by including experts from different levels (micro, meso and macro) and experts from diverse backgrounds in social care data processing and usage in the interviews and reflection session to enrich the desktop study and validate results. Public sources in social care data like the dashboards and monitors used in this study are constantly developing, thus the results of the study are subject to change over time. This research was designed to be a starting point and not to exhaust all options. Different stakeholder settings could generate different results. We have limited the bias by intentionally taking available public indicators as a starting point for stakeholders to reflect on and by including a wide variety of stakeholders in our interviews and the reflection session.

Possible explanations and implications on micro, meso and macro levels

Our results are characteristic for a fragmented health care system (OECD, 2022). In these systems legal and financial silos are a barrier for integrated health care, public health and social care provision governance (Bos, Klazinga, & Kringos, 2021; OECD, 2022). In the Netherlands social care responsibilities have been decentralized from the federal to the municipal level, resulting in variations between municipalities regarding data processing and utilization. There are recent attempts in the policy agenda to integrate care services, expressed by (among other incentives) the Integrated Care Agreement (IZA), the Healthy and Active living Agreement (GALA) and the Housing, Support and Care for the Elderly Agreement (WOZO)[20-23]. To effectively implement and oversee these agreements, actionable information tailored to the right individual at the right time is essential. However, our findings reveal that social care data is fragmented, standardized to a limited extent, and offers suboptimal linkage possibilities with various legal and financial care silos. Despite these challenges, there is a current trend toward increased integration, endorsed by a majority of political parties in the Netherlands. The

For peer review only

outcomes of the November 2023 elections will additionally influence political support for further reforms in financing arrangements aligned with integrated care ambitions.

To effectively monitor and address health system goals, including quality, accessibility, and affordability, it is imperative to link a diverse range of data sources. Statistics Netherlands has demonstrated the feasibility of linkages, although they are frequently employed only at aggregated levels. The recent policy programmes should be accompanied by a monitoring system to monitor progress in achieving the ambitions informing the key stakeholders to support their respective roles. Our findings show that different levels in the system and the different roles represented require different pieces of information in order to support effective decision making. Our findings also indicate that the existing social care data infrastructure falls short in providing individual clients with a comprehensive overview of their integrated health and social care provisions. Additionally, social care providers face challenges in exchanging data with other health and social care professionals. Financers in social care encounter difficulties linking their administrative data to health care domain financers, and the current data infrastructure does not adequately support the monitoring of integrated agreements made between different parties. Recently the Dutch government has been working on an overall strategy and legislation (e.g. Electronic Data Sharing in Health Care Act) on the information infrastructure in health and social care[30, 31]. But the emphasis of the approach is put on the curative health care sector. Establishing a governance structure and guidelines is crucial to facilitate the implementation of actionable linkages across domains and within various legal and financial silos.

Future research

Our research design has provided first-hand insights into the utilization of social care data, addressing a notable knowledge gap in the existing published literature. Policy ambitions to integrate health and social care requires adequate data and information. We recommend further research to identify the essential data and information exchanges required to support integrated health and social care policy goals. The five potential uses found for social care data to improve the governance of integrated care practice and policies could be a focus for further research that supports implementing the desired policies.

Conclusion

This study identified six current uses for social care data: 1) communication and accountability, 2) monitoring social care policy, 3) early warning systems, 4) controls and fraud detection, 5) outreaching

For peer review only

efforts, and 6) prioritization. Further optimization is recommended through standardization, effective management of data exchange across domains, increased awareness of the link between registration and financing, and reinforcing societal trust in data sharing. Additionally, the study revealed five ways in which enhanced social care data could be employed to govern integrated health and social care services: 1) fostering cross-domain learning and cooperation (e.g., through benchmarks), 2) implementing preventative measures and early warning systems, 3) providing insight into the quality and effectiveness of social care from a broader perspective, 4) enhancing accountability of social care toward contracting parties and policy, and 5) enabling a cross-sector data-driven governance model.

Acknowledgements

The authors thank all citizen representatives, health and social care professionals and data experts who were willing to reflect on social data use in the Netherlands so openly during the interviews and the reflection session.

Contributership statement

This research was drafted by VB (Female, BSc, MA; PhD candidate), DK (Female, Associate Professor and Principal Investigator & Educator) and NK (Male, Full Professor of Social Medicine, MD). The desktop research and expert interviews were executed by VB (Experienced in interviews, reflection sessions and qualitative research) in close collaboration and supervision of DK and NK. The reflection session was moderated by NK and accompanied by DK and VB who took notes. All authors have read and approved this manuscript.

Competing interests

No competing interests are declared.

Funding

This work was supported by the European Union Funded Marie Sklodowska-Curie Innovative Training Network "HealthPros" grant number 765141.

Data sharing statement

The data that support the findings of this study are available on request from the corresponding author (VB) for checks on scientific integrity or quality control by licensed parties. For other reasons, due to the personal nature of this data, permission of the participants is required.

For peer review only

Ethics approval

This research proposal has been reviewed and approved by the Medical Ethics Review Committee of the Academic Medical Center prior to data collection (reference number: W22_136 # 22.179).

References

1. Kringos, D., et al., *Managing COVID-19 within and across health systems: why we need performance intelligence to coordinate a global response.* Health Research Policy and Systems, 2020. **18**(1): p. 1-8.
2. World Health Organization, *Monitoring the building blocks of health systems: a handbook of indicators and their measurement strategies*. 2010: World Health Organization.
3. Colombo, F., J. Oderkirk, and L. Slawomirski, *Health Information Systems, Electronic Medical Records, and Big Data in Global Healthcare: Progress and Challenges in OECD Countries.* Handbook of Global Health, 2020: p. 1-31.
4. OECD, *Who Cares? Attracting and Retaining Care Workers for the Elderly*. 2020.
5. OECD, *Realising the Potential of Primary Health Care*. 2020.
6. voor het Regeringsbeleid, W.R., *Kiezen voor houdbare zorg. Mensen, middelen en maatschappelijk draagvlak WRR-Rapport 104.* 2021.
7. World Health Organization, *Strengthening and adjustingpublic health measures throughout the COVID-19 transition phases: policyconsiderations for the WHO European Region, 24 April 2020*. 2020, World Health Organization. Regional Office for Europe.
8. OECD, *Health in the 21st Century*. 2019.
9. OECD, *Health at a Glance 2021*. 2021.
10. Taylor, L.A., et al., *Leveraging the Social Determinants of Health: What Works?* PLoS One, 2016. **11**(8): p. e0160217.
11. Kärki, J. and E. Ailio, *The data model for social welfare in Finland.* Stud Health Technol Inform, 2014. **205**: p. 343-7.
12. Centre, J.R., et al., *Strategic intelligence monitor on personal health systems, phase 3 (SIMPHS3) : Oulu self-care (Finland), case study report*, ed. F. Abadie and C. Codagnone. 2015: Publications Office.
13. Milne, B.J., et al., *Data Resource Profile: The New Zealand Integrated Data Infrastructure (IDI).* Int J Epidemiol, 2019. **48**(3): p. 677-677e.
14. Zhao, J., et al., *Constructing whole of population cohorts for health and social research using the New Zealand Integrated Data Infrastructure.* Aust N Z J Public Health, 2018. **42**(4): p. 382-388.
15. McLeod, K.B., C. Tumen, S. Crichton, S. , *Using Integrated Administrative Data to Identify Youth who are at riskk of poort outcomes as adults.* . 2015, New Zealand Treasury: Wellington.
16. Dixon, S., *The Employment and income effects of eight chronic and acute health conditions*. 2015: New Zealand Treasury Working Paper.
17. de Oliveira, C., et al., *Describing the linkage between administrative social assistance and health care databases in Ontario, Canada.* Int J Popul Data Sci, 2022. **7**(1): p. 1689.
18. Witham, M.D., et al., *Construction of a linked health and social care database resource--lessons on process, content and culture.* Inform Health Soc Care, 2015. **40**(3): p. 229-39.
19. Wastell, D. and S. White, *Making sense of complex electronic records: Socio-technical design in social care.* Applied ergonomics, 2014. **45**(2): p. 143-149.
20. Rijksoverheid, *Integraal Zorg Akkoord*. 2022: Den Haag.
21. Rijksoverheid, *WOZO Programma Wonen, Ondersteuning en Zorg voor Ouderen*. 2022: Den Haag.

For peer review only

22. Rijksoverheid, *Gezond en Actief Leven Akkoord*. 2022: Den Haag.
23. Rijksoverheid, *Programma Toekomstbestendige Arbeidsmarkt Zorg en Welzijn*. 2022: Den Haag.
24. Partners, P., *Verkenning naar domeinoverstijgende samenwerkingsverbanden levert waardevolle inzichten op*, ZonMw, Editor. 2022.
25. Bos, V., N.S. Klazinga, and D.S. Kringos, *Improving performance intelligence for governing an integrated health and social care delivery network: a case study on the Amsterdam Noord district.* BMC Health Serv Res, 2021. **21**(1): p. 517.
26. Kasteridis, P., et al., *Who would most benefit from improved integrated care? Implementing an analytical strategy in South Somerset.* Int J Integr Care, 2015. **15**: p. e001.
27. Society, T.C.o.P.H., *Met de stroom mee: Naar een duurzaam en adaptief stelsel van zorg en ondersteuning*. 2023: The Hague.
28. Rendle, K.A., et al., *Beyond exploratory: a tailored framework for designing and assessing qualitative health research.* BMJ Open, 2019. **9**(8): p. e030123.
29. Van Driesten, G.B., S. Zonderland, S. Kraaijeveld, K. , *Zó werkt het sociaal domein*. 2021: De Argumentenfabriek.
30. Rijksoverheid, *Electronic Data Sharing in Health Care Act*. 2023: The Hague.
31. Ministry of Health, W.a.S., *National Strategy on the Health Information system in the Netherlands*. 2023.

For peer review only

Manuscript – Social care data and its fitness for integrated health and social care service governance: an exploratory qualitative analysis in the Dutch context

Véronique LLC Bos[1,2], Niek S Klazinga[1,2] and Dionne S Kringos[1,2]

[1] Department of Public and Occupational Health, Amsterdam UMC Location University of Amsterdam, Meibergdreef 9, Amsterdam, The Netherlands.

[2] Quality of Care, Amsterdam Public Health research institute, Amsterdam, The Netherlands.

Corresponding author

Véronique Bos

Department of Public and Occupational Health, Amsterdam UMC, University of Amsterdam

Van der Boechorststraat 7, 1081 BT Amsterdam, the Netherlands

Email: v.l.bos@amsterdamumc.nl

Supplemental Material 1 COREQ 32-item checklist

Domain 1: Research team and reflexivity

Personal Characteristics

Nr	Item	Guide question/description	Answer and where to find in manuscript
1	Interviewer/facilitator	Which author/s conducted the interview or focus group?	Interviews: VB<br><br>Reflection session: NK, DK and VB<br><br>Contributorship statement page 19
2	Credentials	What were the researcher's credentials? E.g. PhD, MD	VB: BSc, MA<br><br>NK: Full Professor, MD<br><br>DK: Associate Professor<br><br>Contributorship statement page 19
3	Occupation	What was their occupation at the time of the study?	VB: PhD candidate<br><br>NK: Professor of Social Medicine

For peer review only

			DK: Associate Professor and Principal Investigator & Educator<br><br>Contributorship statement page 19
4	Gender	Was the researcher male or female?	VB: female<br><br>NK: male<br><br>DK: female<br><br>Contributorship statement page 19
5	Experience and training	What experience or training did the researcher have?	Experienced in interviews, reflection sessions and qualitative research<br><br>Contributorship statement page 19

Relationship with participants

Nr	Item	Guide question/description	
6	Relationship established	Was a relationship established prior to study commencement?	Before the start of the study, the study and the research team were introduced to the interviewees and reflection session experts via email.<br><br>Research Methods pages 7-8
7	Participant knowledge of the interviewer	What did the participants know about the researcher? e.g. personal goals, reasons for doing the research	In the communication to participants the background of the interviewer was listed, as well as research goals.<br><br>Research Methods pages 7-8
8	Interviewer characteristics	What characteristics were reported about the interviewer/facilitator? e.g. Bias, assumptions, reasons and interests in the research topic	The credentials, occupation and department of the research team was communicated.<br><br>Research Methods pages 7-8

For peer review only

Domain 2: study design

Theoretical framework

Nr	Item	Guide question/description	
9	Methodological orientation and Theory	What methodological orientation was stated to underpin the study? e.g. grounded theory, discourse analysis, ethnography, phenomenology, content analysis	A constructivism explorative approach with inductive reasoning was used. Research Methods page 6

Participant selection

Nr	Item	Guide question/description	
10	Sampling	How were participants selected? e.g. purposive, convenience, consecutive, snowball	Purposive sampling based on the desktop research and participant referral Research Methods page 7
11	Method of approach	How were participants approached? e.g. face-to-face, telephone, mail, email	The participants were approached via email, phone and LinkedIn. Research Methods page 7
12	Sample size	How many participants were in the study?	18 participants in the interviews, and 10 participants in the reflection session. Results page 9
13	Non-participation	How many people refused to participate or dropped out? Reasons?	3 participants dropped out without stating a reason, 1 participant refused to participate due to prioritisation issues at that moment Research Methods page 7

Setting

Nr	Item	Guide question/description	
14	Setting of data collection	Where was the data collected? e.g. home, clinic, workplace	Most interviews were conducted digitally or in the work environment of the interviewee.

			The reflection session was held digitally.<br><br>Research Methods page 7
15	Presence of non-participants	Was anyone else present besides the participants and researchers?	No<br><br>Contributorship statement page 19
16	Description of sample	What are the important characteristics of the sample? e.g. demographic data, date	Participants from different levels of the health care system (micro, meso, macro) and different perspectives (clients, providers, data processors, policy advisors) were included in the sample.<br><br>Research Methods page 7

Data collection

Nr	Item	Guide question/description	
17	Interview guide	Were questions, prompts, guides provided by the authors? Was it pilot tested?	The interview guideline was grounded in the Health care performance intelligence pyramid and is provided in appendix 3. It was not pilot tested.<br><br>Research Methods page 7
18	Repeat interviews	Were repeat interviews carried out? If yes, how many?	No, however some questions that could not be answered during interviews were answered at a later moment in time or corrected in the transcript by the interviewee.<br><br>Research Methods page 8
19	Audio/visual recording	Did the research use audio or visual recording to collect the data?	Interviews and the reflection session were audio recorded<br><br>Research Methods page 8

For peer review only

20	Field notes	Were field notes made during and/or after the interview or focus group?	Notes were taken by two researchers (VB and DK) during the reflection session.<br><br>Contributor statement page 19
21	Duration	What was the duration of the interviews or focus group?	The duration of the interviews were between 33-60 minutes.<br><br>Research Methods page 7
22	Data saturation	Was data saturation discussed?	Participants could adjust content to their transcript without limitations and the reflection had the function to validate and if needed complement draft findings.<br><br>Research Methods pages 7-8
23	Transcripts returned	Were transcripts returned to participants for comment and/or correction?	Transcripts were emailed to the participants and they were given at least two weeks to review and adjust content to their transcript without limitations.<br><br>Results page 8

Domain 3: analysis and findings

Data analysis

Nr	Item	Guide question/description	
24	Number of data coders	How many data coders coded the data?	One researcher VB drafted the results and the reflection session was used to complement and validate principle findings.<br><br>Contributorship statement page 19
25	Description of the coding tree	Did authors provide a description of the coding tree?	No, a reflection session with experts was used to validate principle findings.<br><br>Research Methods page 8

For peer review only

26	Derivation of themes	Were themes identified in advance or derived from the data?	The transcript pieces were ordered by research question and themes were identified from these grouped transcript pieces.<br><br>Research Methods page 8-9
27	Software	What software, if applicable, was used to manage the data?	Excel was used to order and theme the transcript pieces.<br><br>Research Methods page 8
28	Participant checking	Did participants provide feedback on the findings?	Yes, the reflection session was used to complement and validate draft findings.<br><br>Research Methods page 8

Reporting

Nr	Item	Guide question/description	
29	Quotations presented	Were participant quotations presented to illustrate the themes / findings? Was each<br><br>quotation identified? e.g. participant number	One quote was used anonymously in the article and can be retraced by the researchers via the transcript identification number.<br><br>Results page 15
30	Data and findings consistent	Was there consistency between the data presented and the findings?	Yes, no contradicting statements were made in the interviews.<br><br>Results pages 9-16
31	Clarity of major themes	Were major themes clearly presented in the findings?	There was overall consensus in the reflection session on the final principal findings.<br><br>Results pages 9-16
32	Clarity of minor themes	Is there a description of diverse cases or discussion of minor themes?	In the reflection session examples were added to some statements to highlight nuances.<br><br>Results pages 9-16

For peer review only

Manuscript – Social care data and its fitness for integrated health and social care service governance: an exploratory qualitative analysis in the Dutch context

Véronique LLC Bos[1,2], Niek S Klazinga[1,2] and Dionne S Kringos[1,2]

[1] Department of Public and Occupational Health, Amsterdam UMC Location University of Amsterdam, Meibergdreef 9, Amsterdam, The Netherlands.

[2] Quality of Care, Amsterdam Public Health research institute, Amsterdam, The Netherlands.

Corresponding author

Véronique Bos

Department of Public and Occupational Health, Amsterdam UMC, University of Amsterdam

Van der Boechorststraat 7, 1081 BT Amsterdam, the Netherlands

Email: v.l.bos@amsterdamumc.nl

For peer review only

Supplemental Material 2: Dashboards desktop research & Data sources

Dashboards

Waar staat je gemeente (www.waarstaatjegemeente.nl)

Gemeentezorgspiegel (https://www.vektis.nl/gemeentezorgspiegel)

Regiobeeld (https://www.regiobeeld.nl/)

Labour Market Dashboards (https://www.werk.nl/arbeidsmarktinformatie/dashboards)

Early warning dashboard for possible debt (https://monitorvroegsignaleringshv.nl)

Data sources of included indicators

CBS - Arbeidsdeelname

CBS - Bevolkingsstatistiek

CBS - Bijstandsuitkeringenstatistiek

CBS - Enquête beroepsbevolking

CBS - Enquête sociale samenhang en Welzijn

CBS - Jeugdmonitor

CBS - Gezondheidsmonitor Volwassenen en Ouderen

CBS - Gemeentelijke Monitor Sociaal Domein

CBS - Monitor Abonnementstarief

CBS – Statistiek Re-integratie door Gemeenten

Divosa - Monitor Schuldhulpverlening

Divosa - Monitor Vroegsignalering Schulden

GGD-GHOR

RIVM

Vektis

For peer review only

Manuscript – Social care data and its fitness for integrated health and social care service governance: an exploratory qualitative analysis in the Dutch context

Véronique LLC Bos[1,2], Niek S Klazinga[1,2] and Dionne S Kringos[1,2]

[1] Department of Public and Occupational Health, Amsterdam UMC Location University of Amsterdam, Meibergdreef 9, Amsterdam, The Netherlands.

[2] Quality of Care, Amsterdam Public Health research institute, Amsterdam, The Netherlands.

Corresponding author

Véronique Bos

Department of Public and Occupational Health, Amsterdam UMC, University of Amsterdam

Van der Boechorststraat 7, 1081 BT Amsterdam, the Netherlands

Email: v.l.bos@amsterdamumc.nl

Supplemental Material 3 Translated interview guide

Thank you for your time

Going through informed consent form – when agreed, proceed

Affirm the scope of the research: Wet Maatschappelijke Ondersteuning, Participatiewet, Wet Gemeentelijke Schuldhulpverlening

- What data do you collect?
    - What is the purpose of your data collection?
    - Is there underlying legislation, standardization or other agreements or confinements as to how to manage the data?
- Data input points (potential legislation/standardization at entry point)
    - Who does the entry of the data?
    - With what purpose is the data entered in the system?
    - Is there underlying legislation, standardization or other agreements or confinements as to how to enter the data?
- Data sources and custodians
    - What database is the data collected in and who is owner of the database?
- Interoperability of sources
    - Does the database interact with other databases? If yes, with which other databases?

For peer review only

- Data management responsibilities, stakeholder inclusion, and regulators
  - How is data quality managed?
  - Are stakeholders included in quality management, transferring data to performance indicators, or other ways of data management?
  - What internal/external controls are executed for the database?
- Data outputs (information) and its users
  - What indicators are presented from the database?
  - Who uses the indicators to inform their decision making?
- In your perspective how can we make good use of social care data within the three laws mentioned for the benefit of integrated care? And what are potential hurdles to make use of this data?

For peer review only

Manuscript – Social care data and its fitness for integrated health and social care service governance: an exploratory qualitative analysis in the Dutch context

Véronique LLC Bos[1,2], Niek S Klazinga[1,2] and Dionne S Kringos[1,2]

[1] Department of Public and Occupational Health, Amsterdam UMC Location University of Amsterdam, Meibergdreef 9, Amsterdam, The Netherlands.

[2] Quality of Care, Amsterdam Public Health research institute, Amsterdam, The Netherlands.

Corresponding author

Véronique Bos

Department of Public and Occupational Health, Amsterdam UMC, University of Amsterdam

Van der Boechorststraat 7, 1081 BT Amsterdam, the Netherlands

Email: v.l.bos@amsterdamumc.nl

Supplemental Material 4 Interviewee list with background/expertise

organization	WHY?
VNG GMSD	Custodian of the Municipal Monitor of the Social Domain Dashboard and data.
VNG voorspelmodel	Custodian of the Social Care Act (WMO) prediction model
Divosa	Knowledge organisation supporting municipalities
Municipality of Amsterdam	Data team of the municipality
Stichting Inlichtingenbureau	Data processing service organisation established by the Ministry of Social Affairs and Employment (SZW) processing data about citizens who use social care schemes and facilities.
Vektis	Custodian of the Gemeentezorgspiegel (municipal care dashboard)
De Friesland	Health care insurer in the Friesland region working on integrated care projects using data.
RIVM (Regiobeeld)	Custodian of Regiobeeld

For peer review only

Sociaal Werk Versterkt	Representative of social workers
De Sociale Maatschap	Social care provider in de Amsterdam Noord region
Ketenbureau i-sociaal Domein	Data processing service organisation which facilitates and supports municipalities and healthcare providers in, among other things, simplifying the process from purchasing and tendering to accountability and reducing administrative burdens.
ZorgInstituut	Custodians of the i-standards (e.g. i_WMO standard)
BKR	Custodian credit registry (BKR)
LCR	National representatives for clients in social care

For peer review only

Manuscript – Social care data and its fitness for integrated health and social care service governance: an exploratory qualitative analysis in the Dutch context

Véronique LLC Bos[1,2], Niek S Klazinga[1,2] and Dionne S Kringos[1,2]

[1] Department of Public and Occupational Health, Amsterdam UMC Location University of Amsterdam, Meibergdreef 9, Amsterdam, The Netherlands.

[2] Quality of Care, Amsterdam Public Health research institute, Amsterdam, The Netherlands.

Corresponding author

Véronique Bos

Department of Public and Occupational Health, Amsterdam UMC, University of Amsterdam

Van der Boechorststraat 7, 1081 BT Amsterdam, the Netherlands

Email: v.l.bos@amsterdamumc.nl

Supplemental Material 1 COREQ 32-item checklist

Domain 1: Research team and reflexivity

Personal Characteristics

Nr	Item	Guide question/description	Answer and where to find in manuscript
1	Interviewer/facilitator	Which author/s conducted the interview or focus group?	Interviews: VB<br><br>Reflection session: NK, DK and VB<br><br>Contributorship statement page 19
2	Credentials	What were the researcher's credentials? E.g. PhD, MD	VB: BSc, MA<br><br>NK: Full Professor, MD<br><br>DK: Associate Professor<br><br>Contributorship statement page 19
3	Occupation	What was their occupation at the time of the study?	VB: PhD candidate<br><br>NK: Professor of Social Medicine

For peer review only

			DK: Associate Professor and Principal Investigator & Educator<br><br>Contributorship statement page 19
4	Gender	Was the researcher male or female?	VB: female<br><br>NK: male<br><br>DK: female<br><br>Contributorship statement page 19
5	Experience and training	What experience or training did the researcher have?	Experienced in interviews, reflection sessions and qualitative research<br><br>Contributorship statement page 19

Relationship with participants

Nr	Item	Guide question/description	
6	Relationship established	Was a relationship established prior to study commencement?	Before the start of the study, the study and the research team were introduced to the interviewees and reflection session experts via email.<br><br>Research Methods pages 7-8
7	Participant knowledge of the interviewer	What did the participants know about the researcher? e.g. personal goals, reasons for doing the research	In the communication to participants the background of the interviewer was listed, as well as research goals.<br><br>Research Methods pages 7-8
8	Interviewer characteristics	What characteristics were reported about the interviewer/facilitator? e.g. Bias, assumptions, reasons and interests in the research topic	The credentials, occupation and department of the research team was communicated.<br><br>Research Methods pages 7-8

For peer review only

Domain 2: study design

Theoretical framework

Nr	Item	Guide question/description	
9	Methodological orientation and Theory	What methodological orientation was stated to underpin the study? e.g. grounded theory, discourse analysis, ethnography, phenomenology, content analysis	A constructivism explorative approach with inductive reasoning was used. Research Methods page 6

Participant selection

Nr	Item	Guide question/description	
10	Sampling	How were participants selected? e.g. purposive, convenience, consecutive, snowball	Purposive sampling based on the desktop research and participant referral Research Methods page 7
11	Method of approach	How were participants approached? e.g. face-to-face, telephone, mail, email	The participants were approached via email, phone and LinkedIn. Research Methods page 7
12	Sample size	How many participants were in the study?	18 participants in the interviews, and 10 participants in the reflection session. Results page 9
13	Non-participation	How many people refused to participate or dropped out? Reasons?	3 participants dropped out without stating a reason, 1 participant refused to participate due to prioritisation issues at that moment Research Methods page 7

Setting

Nr	Item	Guide question/description	
14	Setting of data collection	Where was the data collected? e.g. home, clinic, workplace	Most interviews were conducted digitally or in the work environment of the interviewee.

For peer review only

			The reflection session was held digitally.<br><br>Research Methods page 7
15	Presence of non-participants	Was anyone else present besides the participants and researchers?	No<br><br>Contributorship statement page 19
16	Description of sample	What are the important characteristics of the sample? e.g. demographic data, date	Participants from different levels of the health care system (micro, meso, macro) and different perspectives (clients, providers, data processors, policy advisors) were included in the sample.<br><br>Research Methods page 7

Data collection

Nr	Item	Guide question/description	
17	Interview guide	Were questions, prompts, guides provided by the authors? Was it pilot tested?	The interview guideline was grounded in the Health care performance intelligence pyramid and is provided in appendix 3. It was not pilot tested.<br><br>Research Methods page 7
18	Repeat interviews	Were repeat interviews carried out? If yes, how many?	No, however some questions that could not be answered during interviews were answered at a later moment in time or corrected in the transcript by the interviewee.<br><br>Research Methods page 8
19	Audio/visual recording	Did the research use audio or visual recording to collect the data?	Interviews and the reflection session were audio recorded<br><br>Research Methods page 8

For peer review only

20	Field notes	Were field notes made during and/or after the interview or focus group?	Notes were taken by two researchers (VB and DK) during the reflection session.<br><br>Contributor statement page 19
21	Duration	What was the duration of the interviews or focus group?	The duration of the interviews were between 33-60 minutes.<br><br>Research Methods page 7
22	Data saturation	Was data saturation discussed?	Participants could adjust content to their transcript without limitations and the reflection had the function to validate and if needed complement draft findings.<br><br>Research Methods pages 7-8
23	Transcripts returned	Were transcripts returned to participants for comment and/or correction?	Transcripts were emailed to the participants and they were given at least two weeks to review and adjust content to their transcript without limitations.<br><br>Results page 8

For peer review only

Domain 3: analysis and findings

Data analysis

Nr	Item	Guide question/description	
24	Number of data coders	How many data coders coded the data?	One researcher VB drafted the results and the reflection session was used to complement and validate principle findings.<br><br>Contributorship statement page 19
25	Description of the coding tree	Did authors provide a description of the coding tree?	No, a reflection session with experts was used to validate principle findings.<br><br>Research Methods page 8

26	Derivation of themes	Were themes identified in advance or derived from the data?	The transcript pieces were ordered by research question and themes were identified from these grouped transcript pieces.<br><br>Research Methods page 8-9
27	Software	What software, if applicable, was used to manage the data?	Excel was used to order and theme the transcript pieces.<br><br>Research Methods page 8
28	Participant checking	Did participants provide feedback on the findings?	Yes, the reflection session was used to complement and validate draft findings.<br><br>Research Methods page 8

Reporting

Nr	Item	Guide question/description	
29	Quotations presented	Were participant quotations presented to illustrate the themes / findings? Was each<br><br>quotation identified? e.g. participant number	One quote was used anonymously in the article and can be retraced by the researchers via the transcript identification number.<br><br>Results page 15
30	Data and findings consistent	Was there consistency between the data presented and the findings?	Yes, no contradicting statements were made in the interviews.<br><br>Results pages 9-16
31	Clarity of major themes	Were major themes clearly presented in the findings?	There was overall consensus in the reflection session on the final principal findings.<br><br>Results pages 9-16
32	Clarity of minor themes	Is there a description of diverse cases or discussion of minor themes?	In the reflection session examples were added to some statements to highlight nuances.<br><br>Results pages 9-16

For peer review only
